# Generalized correlation coefficient for genome-wide association analysis of cognitive ability in twins

**DOI:** 10.18632/aging.104198

**Published:** 2020-11-24

**Authors:** Afsaneh Mohammadnejad, Marianne Nygaard, Shuxia Li, Dongfeng Zhang, Chunsheng Xu, Weilong Li, Jesper Lund, Lene Christiansen, Jan Baumbach, Kaare Christensen, Jacob v. B. Hjelmborg, Qihua Tan

**Affiliations:** 1 Epidemiology, Biostatistics and Biodemography, Department of Public Health, University of Southern Denmark, Odense, Denmark; 2 Department of Epidemiology and Health Statistics, School of Public Health, Qingdao University, Qingdao, China; 3 Qingdao Center for Disease Control and Prevention, Qingdao, China; 4 Department of Clinical Immunology, Copenhagen University Hospital, Rigshospitalet, Copenhagen Ø, Denmark; 5 Computational Biomedicine, Department of Mathematics and Computer Science, University of Southern Denmark, Odense, Denmark; 6 Experimental Bioinformatics, TUM School of Life Sciences, Technical University of Munich, Munich, Germany; 7 Unit of Human Genetics, Department of Clinical Research, University of Southern Denmark, Odense, Denmark

**Keywords:** generalized correlation coefficient, GWAS, cognition, twins

## Abstract

Despite a strong genetic background in cognitive function only a limited number of single nucleotide polymorphisms (SNPs) have been found in genome-wide association studies (GWASs). We hypothesize that this is partially due to mis-specified modeling concerning phenotype distribution as well as the relationship between SNP dosage and the level of the phenotype. To overcome these issues, we introduced an assumption-free method based on generalized correlation coefficient (GCC) in a GWAS of cognitive function in Danish and Chinese twins to compare its performance with traditional linear models. The GCC-based GWAS identified two significant SNPs in Danish samples (rs71419535, p *=* 1.47e-08; rs905838, p = 1.69e-08) and two significant SNPs in Chinese samples (rs2292999, p = 9.27e-10; rs17019635, p = 2.50e-09). In contrast, linear models failed to detect any genome-wide significant SNPs. The number of top significant genes overlapping between the two samples in the GCC-based GWAS was higher than when applying linear models. The GCC model identified significant genetic variants missed by conventional linear models, with more replicated genes and biological pathways related to cognitive function. Moreover, the GCC-based GWAS was robust in handling correlated samples like twin pairs. GCC is a useful statistical method for GWAS that complements traditional linear models for capturing genetic effects beyond the additive assumption.

## INTRODUCTION

Cognitive function is an important phenotype involving multiple mental abilities including learning, thinking, reasoning, remembering, problem-solving, decision-making, and attention. Thus, cognitive function affects health and quality of life in the elderly. Recent twin and family studies have shown that the heritability of general cognitive function is more than 50% in adolescence through adulthood to older age [1], suggesting a substantial genetic contribution to the phenotype. Despite the relatively large number of genome-wide association studies (GWASs) of cognition performed to date, a lot of the heritability is still unexplained. For instance, Savage et al., 2018 [2] reported the largest GWAS of intelligence to date (N = 269,867), identifying 205 significantly associated genomic loci and h^2^_*SNP*_ = 0.22. In addition, different limitations in current GWASs might be the reason for the situation due to the distribution of cognitive measurements as well as the complex relationship between SNP genotypes and cognitive performance. One of the assumptions in the popular linear models used for GWAS is normality of the phenotype of interest. Unfortunately, the distribution of cognitive measurements is typically skewed [3]. Also, the additive genetic effect has been the most popular assumption in current genetic association studies and the only genetic model addressed in studies using linear models. However, the pattern of genetic associations is likely more complex, including both linear (additive) and non-linear (non-additive) relationships [4, 5].

As a recent development, the generalized measure of dependency for analysis of omics data has been proposed [4–6]. The concept of a generalized measure of association comes from the rank correlation and information theory. The rank correlation was defined by Hoeffding’s D by measuring the difference between the joint rank of two random variables *x* and *y* and the product of their marginal rank [7]. In 2012, Reshef [8] and his colleagues proposed the maximal information coefficient (MIC), which belongs to a larger class of maximal information-based nonparametric exploration (MINE), as a measure of association. MIC is able to not only measure two-variable dependence by calculating a score that is approximately equal to a coefficient of determination (R^2^) relative to the regression function, but also to detect non-linear relationships between variables. Recently, Murrell et al., 2016 [9] proposed an approach similar to MIC that additionally included the advantages of fast convergence with rising sample size and having more power to detect the relationships compared to MIC. Both MIC and Murrell’s method follow desired properties of bivariate association, generality and equitability [9]. Murrell and his colleagues have suggested a new measure of bivariate association, which is the same as R^2^, but powerful when the parametric form of association is unknown. A point of particular importance is that through applying this method both generality and equitability can be attained by estimating a generalized R^2^ based on density approximation. We believe that this method has advantages that merit it to be considered in GWAS as the associations between the SNP dosage and the phenotype are expected to be complex. More importantly, this method does not rely on strict assumptions such as normality of the phenotype distribution as well as linearity of the genetic effect (or an additive genetic effect, which is assumed in most GWAS). Additionally, when related samples such as twins or family data are included in association studies, more model assumptions are imposed. Hence, the assumption-free or generalized measurements of association may be advantageous in genetic association studies.

In this study, we aimed to compare the performance of popular linear regression models and the generalized correlation coefficient (GCC) method to identify genetic variants, genes and pathways in Danish and Chinese GWASs of cognitive function.

## RESULTS

The study population comprised Danish and Chinese GWAS data. A description of the two samples is illustrated in Table 1. The means of cognitive function in Danish and Chinese data are 45.86 and 21.01 respectively. We validated the three models, GCC, Kinship, and mixed-linear model (LME), by estimating type I error rates after simulating genotypes for one SNP and a random phenotype (standard normal) for 1000 replications. The type I error rates estimated for GCC, Kinship and LME were 0.052, 0.052 and 0.050, respectively. This indicates that the three models are generally unbiased.

**Table 1:** Descriptive statistics of the 900 dizygotic twins and 272 single monozygotic twins included in the Danish GWAS and the 278 dizygotic twins included in the Chinese GWAS.

**GWAS Variables**	**Male**	**Female**	**Total**
Danish sample	611	561	1172
Mean of age± sd (min, max)	66.51±6.05(56.4,79.88)	66.12±5.93(55.94,80.21)	66.32±6.0(55.94,80.21)
Mean of cognitive score± sd (min, max)	45.03±9.83(11.68,84.93)	46.74±9.94(21.16,83.69)	45.86±9.91(11.68, 84.93)
Chinese sample	141	137	278
Mean of age± sd (min, max)	51.04±7.04(41,70)	51.18±7.04(40,70)	51.11±7.03(40,70)
Mean of cognitive score± sd (min, max)	20.59±4.65(5,30)	21.44±4.77(5,29)	21.01±4.72(5,30)

### Generalized correlation coefficient and linear models

**Figure 1:**
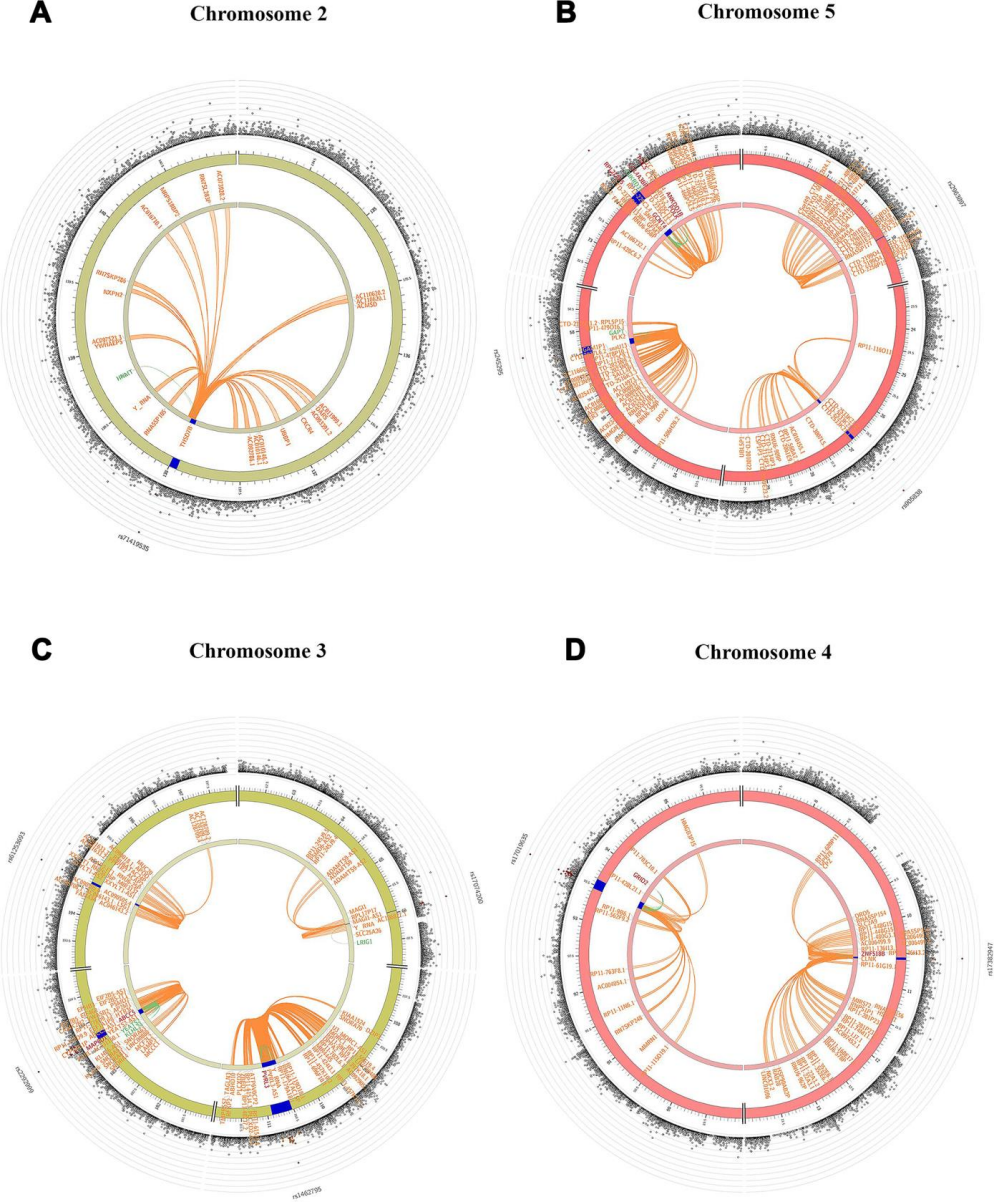
Circos plots indicating genes from genome-wide significant SNPs on chromosomes 2 (**A**), 5 (**B**) in Danish sample and 3 (**C**), 4 (**D**) in Chinese sample based on GCC model. The blue region shows the genomic risk region. Genes mapped by chromatin interaction, eQTL and both are displayed in orange, green and red respectively. The most outer layer shows a Manhattan plot only for SNPs with p < 0.05 and SNPs are colored in red based on linkage disequilibrium (LD) patterns with the lead SNPs.

**Figure 2:**
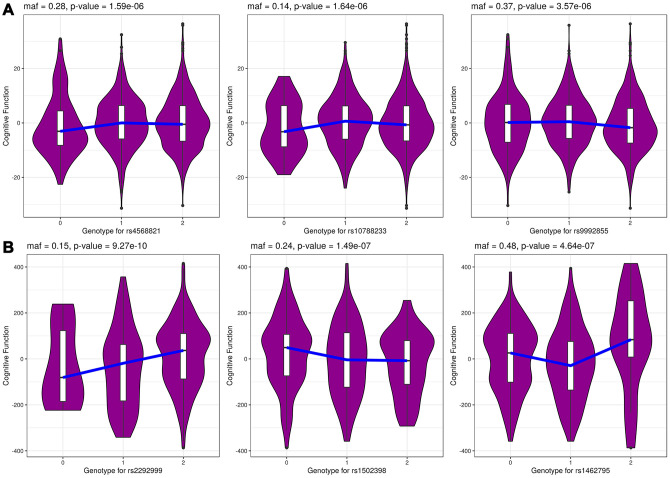
****The genotype-specific density distribution for top 3 genotyped SNPs in Danish (**A**) and Chinese (**B**) samples from GCC model. The x-axis shows the SNP genotypes 0, 1 and 2 and y-axis shows cognitive function phenotype.

**Figure 3:**
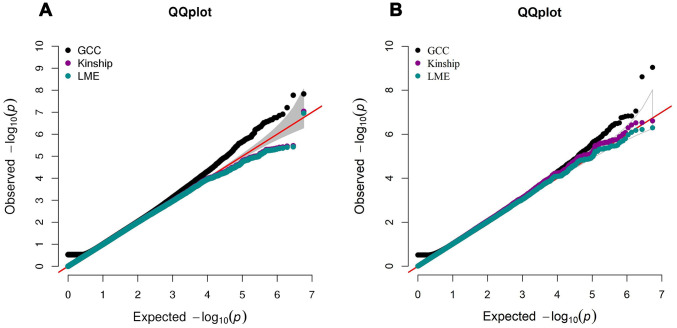
****QQ plot comparing the performance of GCC, kinship and LME models in Danish (**A**) and Chinese (**B**) GWAS data. The left QQ plot is from Danish sample and the right QQ plot is from Chinese sample. In each plot, x-axis is the expected p-value and y-axis is the observed p-value from the GWAS.

**Figure 4:**
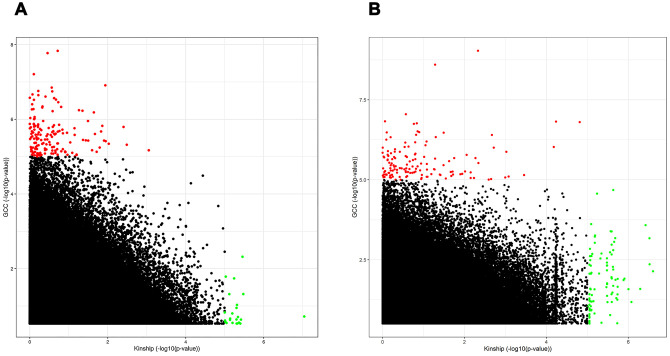
****Scatter plot comparing the performance of SNPs in linear model to the GCC model in both Danish (**A**) and Chinese (**B**) samples. Th x-axis and y-axis show -log10(p-value) from Kinship and GCC models respectively.

The summary results for the top 30 SNPs from the GWASs performed in Danish and Chinese samples using each of the three models are shown in Supplementary Tables 1–6. The use of the GCC method resulted in the identification of two genome-wide significant (p < 5e-8) SNPs in Danish samples: rs71419535 (p *=* 1.47e-08) on chromosome 2 near the gene *THSD7B*, and rs905838 (p = 1.69e-08) on chromosome 5 near the gene *CTD-2533K21.4*; and two genome-wide significant SNPs in Chinese samples: rs2292999 (p *=* 9.27e-10) on chromosome 3 near the gene *ABCC5*, and rs17019635 (p = 2.50e-09) on chromosome 4 near the gene *GRID2*. Circos plots for the genome-wide significant SNPs identified using GCC are shown in Figure 1. In contrast, no genome-wide significant SNPs were identified in either the Danish or Chinese sample when using Kinship or LME. The density plots of the three genotyped SNPs (p < 1e-06) in the Danish and Chinese GWAS when using GCC are depicted in Figure 2. In general, if genotype and cognition are independent, the widest parts of the density beans should be on the same level (height), around 0 on the Y-axis. The patterns in Figure 2 show clear deviations from independence as well as from an additive genetic effect. The QQ plots comparing the three models in both studies are shown in Figure 3A (Danish) and 3B (Chinese). Across the three models, GCC was able to detect non-random SNPs as compared with the kinship and LME models. The genomic inflation factors for GCC, Kinship and LME were 1.061, 0.995 and 0.998 for the Danish sample and 1.059, 1.013 and 1.033 for the Chinese sample. Supplementary Figure 1 shows QQ plots comparing the GCC GWAS results with the GWAS results obtained using each of the linear models. Both plots show a higher efficiency of GCC than of kinship and LME. Figure 4 compares SNP p-values from the GCC GWAS with SNP p-values from the kinship model GWAS in Danish (4A) and Chinese (4B) samples, with suggestively significant SNPs (p <1e-05) colored red if identified using GCC and green if identified using the kinship (linear) model. Similar comparisons of the GCC GWAS results with those of the LME model GWAS are shown in Supplementary Figure 2. Manhattan plots for all three models in both samples are shown in Supplementary Figure 3.

### Post-GWAS analysis in FUMA

For the GCC GWAS results in the Danish and Chinese samples, the post-GWAS analysis in FUMA identified 29 and 24 independent SNPs with p < 1e-6, 862 and 618 mapped genes, as well as 180 and 204 tagged SNPs that are in linkage disequilibrium (LD) (r^2^ < 0.6) with the independent significant SNPs, respectively. Most of the tagged SNPs identified in FUMA from the Danish GWAS results are positioned in intergenic and ncRNA intronic regions (Supplementary Figure 4). Similarly, most of the tagged SNPs identified from the Chinese GWAS results are positioned in intergenic and intronic regions, but with a smaller proportion in downstream regions (Supplementary Figure 5). In contrast, most of the tagged SNPs identified from the Kinship and LME GWAS results are in ncRNA-intronic and intergenic regions (Supplementary Figures 6–8).

### Gene-based test in MAGMA

The MAGMA gene-set analysis was performed for all SNPs based on the results of the GWAS using each of the three modules. In the Danish and Chinese samples, respectively, 1115/18787 ≈ 5.93% and 1024/18918 ≈ 5.41% of genes from the GCC GWAS, 1046/18787 ≈ 5.57% and 1069/18918 ≈ 5.65% of genes from the kinship model GWAS, and 1034/18787 ≈ 5.50% and 1014/18918 ≈ 5.36% of genes from the LME model GWAS were identified with a p < 0.05.

**Figure 5:**
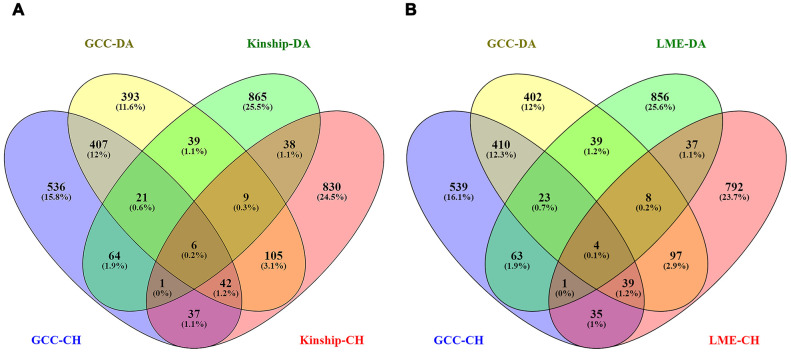
The Venn diagrams showing the number of overlapped genes with p < 0.05 among GCC and Kinship models from both samples in left plot (**A**) and GCC and LME models in the right plot (**B**). The total number of genes included are GCC-DA (GCC in Danish data): 1115, GCC-CH (GCC in Chinese data): 1024, Kinship-DA (Kinship in Danish data): 1046, Kinship-CH (Kinship in Chinese data): 1069, LME-DA (LME in Danish data): 1034 and LME-CH (LME in Chinese data): 1014.

Among the genes identified with a p < 0.05 in the Danish sample, a total of 476, 50 and 54 genes were replicated in the Chinese sample in GCC GWAS results, LME model GWAS results, and Kinship model GWAS results respectively. A Venn diagram showing the number of overlapping genes for both Danish and Chinese samples is shown in Figure 5A, 5B.

The two top genes identified from the discovery data GCC GWAS results were *CSMD1* (p = 5.63e-07, FDR = 0.01) and *PTPRD* (p = 1.68e-5, FDR = 0.16), which were both replicated in the Chinese data (Supplementary Table 7, 8). The two top genes identified from the Kinship model and LME model GWAS results in the Danish sample were *MNT* (Kinship model p = 1.70e-5, LME model p = 1.79e-5) and *MS4A2* (Kinship model p = 8.3-e5, LME model p = 8.69-e5), while the two top genes identified in the Chinese sample were *TEX26* (Kinship model p = 2.21e-7, LME model p = 4.12e-6) and *WDPCP* (Kinship model p = 1.13e-6, LME model p = 1.83e-6).

### Biological pathway analysis

Finally, the results of the overrepresentation analysis performed in GSEA based on genes with p < 0.05 in the gene-based analysis are shown in Supplementary Tables 9–15. The Significant (FDR < 0.05) KEGG pathways identified in the GSEA analysis for genes with p < 0.05 found to overlap in gene-based analyses of both GCC and linear model results are illustrated in Table 2. By using genes with p < 0.05 overlapping in the GCC results from the two samples, 28 KEGG pathways with FDR < 0.05 were found (Supplementary Table 15). In contrast, the 54 and 50 replicated genes from the Kinship and LME analyses were not adequate for pathways analysis.

**Table 2:** Significant KEGG pathways (FDR< 0.05) overlapping between GCC, Kinship model and LME model GWAS results from both Danish and Chinese samples.

**Model**	**Gene set name**	**Description**	**P-value**	**FDR q-value**
GCC-DA-CH^*^	KEGG_PATHWAYS_IN_CANCER	Pathways in cancer	1.08e-12	2.01e-10
GCC-DA-CH	KEGG_AXON_GUIDANCE	Axon guidance	1.16e-10	1.08e-08
GCC-DA-CH	KEGG_ARRHYTHMOGENIC_RIGHT_VENTRICULAR_CARDIOMYOPATHY_ARVC	Arrhythmogenic right ventricular cardiomyopathy (ARVC)	2.27e-09	1.41e-07
GCC-DA-CH	KEGG_VASCULAR_SMOOTH_MUSCLE_CONTRACTION	Vascular smooth muscle contraction	4.67e-09	2.17e-07
GCC-DA-CH	KEGG_CALCIUM_SIGNALING_PATHWAY	Calcium signaling pathway	8.07e-09	3e-07
GCC-DA-CH	KEGG_FOCAL_ADHESION	Focal adhesion	1.51e-08	4.67e-07
GCC-DA-CH	KEGG_MAPK_SIGNALING_PATHWAY	MAPK signaling pathway	2.35e-07	6.26e-06
GCC-DA-CH	KEGG_HYPERTROPHIC_CARDIOMYOPATHY_HCM	Hypertrophic cardiomyopathy (HCM)	6.33e-07	1.47e-05
GCC-DA-CH	KEGG_DILATED_CARDIOMYOPATHY	Dilated cardiomyopathy	1.64e-06	3.04e-05
GCC-DA-CH	KEGG_LONG_TERM_DEPRESSION	Long-term depression	4.43e-06	7.5e-05
GCC-DA-CH	KEGG_GNRH_SIGNALING_PATHWAY	GnRH signaling pathway	6.09e-06	9.44e-05
GCC-DA-CH	KEGG_ADHERENS_JUNCTION	Adherens junction	6.75e-06	9.65e-05
GCC-DA-CH	KEGG_BASAL_CELL_CARCINOMA	Basal cell carcinoma	2.34e-05	0.00029
GCC-DA-CH	KEGG_HEDGEHOG_SIGNALING_PATHWAY	Hedgehog signaling pathway	2.72e-05	0.000294
GCC-DA-CH	KEGG_REGULATION_OF_ACTIN_CYTOSKELETON	Regulation of actin cytoskeleton	4.39e-05	0.000409
GCC-DA-CH	KEGG_GAP_JUNCTION	Gap junction	5.08e-05	0.00045
GCC-DA-CH	KEGG_PHOSPHATIDYLINOSITOL_SIGNALING_SYSTEM	Phosphatidylinositol signaling system	5.87e-05	0.000496
GCC-DA-CH	KEGG_CELL_ADHESION_MOLECULES_CAMS	Cell adhesion molecules (CAMs)	0.000116	0.000896
GCC-DA-CH	KEGG_ECM_RECEPTOR_INTERACTION	ECM-receptor interaction	0.000138	0.000989
GCC-DA-CH	KEGG_TYPE_II_DIABETES_MELLITUS	Type II diabetes mellitus	0.000347	0.00239
GCC-DA-CH	KEGG_MELANOGENESIS	Melanogenesis	0.000624	0.00387
Kin-LME-DA-CH	KEGG_FOCAL_ADHESION	Focal adhesion	0.000777	0.0361
Kin-LME-DA-CH**	KEGG_WNT_SIGNALING_PATHWAY	Wnt signaling pathway	0.001980	0.0462
Kin-LME-DA-CH	KEGG_T_CELL_RECEPTOR_SIGNALING_PATHWAY	T cell receptor signaling pathway	0.002090	0.0462

*GCC-DA-CH: Pathways overlapping in GCC GWAS results from both Danish (DA) and Chinese (CH) samples.

**Kin-LME-DA-CH: Pathways overlapping in Kinship model and LME model GWAS results from both Danish and Chinese samples.

## DISCUSSION

The literature on GWAS data analysis has been dominated by multiple assumptions (e.g. additive or dominant genetic effects, and normal distribution of phenotypes), which, if not fulfilled, could be speculated to be responsible for low statistical power, missing heritability and the lack of replication of GWAS findings. Our study, through model comparison and validation of GWAS results from two independent samples of different ethnicity, illustrates the strength of the GCC method over popular linear models, with more replicated GCC-based results and cognition-implicated SNPs and functional pathways.

Among the two genome-wide significant SNPs identified in the GCC-based GWAS in the Danish sample, rs71419535 is near *THSD7B*, which is associated with melanoma, metabolism, and Cutaneous Malignant 1 diseases. Variation in this gene has been shown to associate with multiple system atrophy, which is an adult-onset neurodegenerative disorder [10]. The other SNP, rs905838, is near *CTD-2533K21.4*, which is a novel transcript highly expressed in brain hippocampus. Among the two genome-wide significant SNPs found in the GCC-based GWAS in the Chinese sample, rs2292999 on chromosome 3 is located near *ABCC5*, which is expressed in both brain and muscle tissue, and rs17019635 on chromosome 3 near *GRID2*, which is expressed in the brain. The *ABCC5* gene has previously been found to encode a general glutamate conjugate and analog transporter that can limit the brain levels of endogenous metabolites, drugs, and toxins [11]. *ABCC5* is additionally related to the ‘Blood-Brain Barrier’ and ‘Immune Cell Transmigration’ pathways. Glutamate is the principal excitatory neurotransmitter in the brain. It is crucial for learning and memory in everyday brain functions but causes excitotoxic damage in traumatic brain injury and stroke [11, 12]. The *GRID2* gene is highly expressed in brain and associated with depression. This gene plays a role in synapse organization between parallel fibers and Purkinje cells (a class of GABAergic neurons in the cerebellum in the brain). The implications of GABAergic neurotransmission in Alzheimer’s disease (AD) have been discussed [13–15], and in the processes of learning and memory, changes in GABAergic function could be an important factor in both early and later stages of AD pathogenesis [15]. Furthermore, the two top genes from the GCC-based GWAS found in both the Danish and Chinese samples, *CSMD1* and *PTPRD*, are very interesting; a variant in *CSMD1* was recently associated with cognitive function [16], and in a very recent study, the *PTPRD* gene was discussed as a druggable target, and a marker for and an important constituent of brain circuits of likely importance for major brain-based phenotypes [17].

Although some significant pathways were identified from the GWAS results generated using all models, more biologically meaningful pathways were found when using the GCC method. Importantly, more genes and pathways identified from the CGG GWAS results were replicated in the Chinese sample than was the case for the linear models. Some replicated pathways from the GCC model GWAS results were pathways in calcium signaling, Type II diabetes mellitus, long-term depression, melanogenesis, axon guidance, focal adhesion, MAPK-signaling, Hedgehog signaling, GnRH signaling, and some related to cardiomyopathy (Table 2). The replicated pathways from the linear model GWAS results were related to focal adhesion, Wnt signaling, and T cell receptor signaling (Table 2).

Calcium signaling and potassium channels have been reported in aging-related diseases, including cognitive function. Calcium signaling is a crucial messenger between synapse and nucleus as calcium transient in nucleus is needed for neuroadaptations to switch on the necessary genes [18]. Additionally, dysregulated expression of calcium signaling genes has been shown to occur with progression of Alzheimer’s-type pathology in the aging brain [19]. Type II diabetes mellitus is discussed in numerous studies because of the presumed role of it in increasing the risk of cognitive impairment and dementia [20, 21]. Focal adhesion is involved in integrin adhesion, communication between the extracellular matrix and the actin cytoskeleton, and the regulation of many cell types. Loss of cell adhesion can lead to cell death and altered focal signaling has been linked to synaptic loss, which may cause AD [22, 23]. There is evidence that axon guidance might play a role in some brain disorders such as Parkinson’s and AD [24].

The Wnt signaling pathway is also important and many recent studies have discussed and reported this pathway in relation to aging and AD, where the loss of canonical Wnt signaling has been found to be involved in the pathogenesis of AD, and play a role in synaptic plasticity and maintenance in the adult brain [25, 26].

In the literature, there is evidence regarding the role of MAPK-signaling, long-term depression, Hedgehog signaling, GnRH signaling, melanogenesis, T cell receptor pathways, cancer, vascular smooth muscle cells and hypertrophic cardiomyopathy (HCM) on cognitive function, Alzheimer’s, Parkinson's and Huntington’s diseases, and age-related neurodegenerative disorders [19, 27–34].

Moreover, worth mentioning are the pathways ‘neuroactive ligand receptor interaction’ and ‘Alzheimer's disease’ that were identified among the 28 KEGG pathways identified from the 476 genes overlapping between the gene-based analysis of Danish and Chinese GCC-based GWAS results (Supplementary Table 15). The neuroactive ligand receptor interaction is important as it comprises genes involved in transmission across chemical synapses [19], and cognitive decline is the earliest sign of AD.

In Figure 4, it is indicated that the statistical significance or the p-values for the same SNPs identified by the GCC and the kinship models are very different. This can potentially be explained by the fact that the linear model is unable to capture SNPs with non-additive effects, while SNPs with an additive effect are best fitted by the linear model. Based on this, we believe that to detect SNPs with an additive effect and SNPs with a non-additive effect both the GCC and a linear model should be applied on the same data when performing a GWAS, i.e. with GCC as a complementary approach to the conventional linear model. As shown in Figure 4, the number of SNPs with a non-additive effect is relatively large compared to the number of SNPs with an additive effect in both the Danish and Chinese samples. This finding is highly important because it suggests that a considerable number of significant SNPs could have been missed in GWASs performed so far due to the search being limited to SNPs with an additive genetic effect.

A limitation of the GCC method, and different from the conventional linear models that report regression coefficients with a direction of effect (+ or -), is that the association parameter from GCC, A, has no direction, which is understandable because direction of effect does not make sense in case of a non-linear relationship. We argue that, as a hypothesis-free or agnostic approach, the primary goal is to identify reliably significant SNP markers. The pattern of association for specific markers can be examined empirically by plotting the data to reveal the various relationships not limited to minus or plus. For SNPs not captured by the linear model, the linear model results can still provide reference information about the direction of the GCC correlation. However, as an assumption-free approach, GCC is inherently robust in handling correlated or structured samples in association testing. The QQ plots in Figure 3 all show that the statistical significance estimated by GCC is not affected by the twin correlation in our samples, with genomic inflation factors comparable to those found for kinship and mixed effect models, which are models specifically dealing with the twin correlation in our samples. In fact, the null distribution for estimating GCC is calculated based on the marginal distributions of the correlated twin samples. As a result, the association assessment by GCC is conditional on the correlated structure in the twin samples, ensuring unbiased estimates of statistical significance. Moreover, unlike the linear models, GCC is insensitive to outliers or extreme phenotype values. All of the above features make GCC a valuable approach for GWAS of complex phenotypes.

## CONCLUSIONS

As a generalized measurement of association, GCC is capable of capturing different patterns of genotype-phenotype correlation, not limited to additive genetic effects. As an assumption-free approach, GCC is robust in dealing with correlated samples, e.g. twins, and skewed phenotype distributions, e.g. cognition, as compared to the linear models. By exemplifying and illustrating the advantages and the necessity of GCC in GWAS, this study could serve as a precedent for application and further development of assumption-free methods to complement current GWAS on cognitive traits and on other complex traits as well.

## MATERIALS AND METHODS

### Participants and cognitive measurements

The Danish sample comprises 1172 participants aged 50-80 years, including 440 same-sex dizygotic (DZ) (238 male, 202 female) twins, 460 (230 male, 230 female) opposite-sex DZ twins and 272 (143 male, 129 female) single monozygotic (MZ) twins recruited by the Danish Twin Registry as a part of the study of Middle-Aged Danish Twins (MADT) (https://pubmed.ncbi.nlm.nih.gov/31544734/). General cognitive functioning was assessed by a cognitive composite score computed from five brief cognitive tests evaluating verbal fluency, attention and working memory (digits forward and digits backward), and memory (immediate and delayed word recall) [35]. Scores obtained from each cognitive test were first standardized using the means and standard deviations of the MADT participants who were 46-50-year-old born from 1949-1952, and then the 5 scores were summed to obtain the composite score. If individuals had two or more scores missing, the cognitive composite score was coded as not available. If individuals had just one missing score, their composite score was computed by prorating the 4 scores they had (i.e., by multiplying by 5/4) [36] (Table 1). The 5 test scores were given equal weight and at most one missing item was allowed. The final cognitive composite score was standardized to a mean of 50 and an SD of 10. For the Danish sample, written informed consent was obtained from all participants and the study was approved by the Regional Committees on Health Research Ethics for Southern Denmark (S-VF-19980072).

Participants in the Chinese sample were 278 dizygotic (DZ) twins aged 40-70 years, including 41 male, 39 female, and 59 opposite sex twin pairs, who completed the Montreal Cognitive Assessment (MoCA) questionnaire to measure their cognitive function and had blood samples taken for DNA extraction and genotyping (Table 1). All voluntary twin subjects gave their informed consent and then completed the Montreal Cognitive Assessment (MoCA) questionnaire and physical examination under the supervision of physicians at the Qingdao CDC. Each twin pair was interviewed face to face by the same well-trained and experienced investigator. Details about sample collection and ethical approval have been described elsewhere [37]. For the Chinese sample, ethical approval was obtained from the Institutional Review Boards of Qingdao Center for Disease Control and Prevention (Qingdao CDC). The study was performed in accordance with the ethical principles of the Helsinki Declaration. All individuals provided a written consent prior to completing the questionnaire and venous blood collection for DNA extraction at Qingdao CDC.

### Genotyping and SNP imputation

Genome-wide SNP genotyping for all the twins in Danish data was conducted using Illumina PsychArray (Illumina, San Diego, California, USA). Genotyping was conducted by the SNP&SEQ Technology Platform, Science for Life Laboratory, Uppsala, Sweden (http://snpseq.medsci.uu.se/genotyping/snp-services/). Pre-imputation quality control (QC) removed SNPs on genotype call rate < 98%, Hardy-Weinberg equilibrium (HWE) p < 10^-6^, and MAF = 0, and individuals on sample call rate < 99%, relatedness and gender mismatch. Pre-phasing and imputation to the 1000 Genomes phase 3 reference panel was performed using IMPUTE2 [2]. The Chinese data genotyping was implemented using the Illumina Infinium Omni2.5Exome-8v1.2BeadChip platform (Illumina, San Diego, California, USA) which contains 2,608,742 SNPs. Before imputation, QC was conducted in PLINK 2 [38] to remove SNPs with a minor allele frequency (MAF) < 0.05, a HWE p < 1e-4, a call-rate < 0.98 as well as SNPs with strand issues. Imputation was done via the Michigan imputation server [39], using the 1000 Genomes phase 3 reference panel. In post-imputation QC for both datasets, SNPs were filtered according to the following thresholds: 1) a minimum imputation INFO score (information metric) of > 0.6, and 2) a MAF of at least 5%. This resulted in 5,779,266 and 5,432,814 genotyped and imputed SNPs to be included in the statistical analysis in the Danish and Chinese samples, respectively. Only autosomal SNPs were analyzed in both studies.

### Statistical analysis

### Generalized correlation coefficient and parametric linear models

We performed the association test of cognitive scores with imputed allele dosages using the mixed-effect kinship model (Kinship) from the Kinship2 package [40], the mixed-linear model (LME) from the *lme4* package [41], and the GCC model from the matie package [9] in R. In the association tests, the cognitive measurements were adjusted for age and sex. Normality of the cognitive score phenotypes is not a requirement in GCC analysis, whereas it is crucial in both linear methods. Hence, in the Chinese data we used Box-Cox transformation to ensure the normality of data for all three models in order to make an unbiased comparison among the models. The kinship model calculates a kinship matrix and integrates it in the covariance matrix of the genetic data, whereas the linear mixed-effect model corrects for the twin correlation in the sample by including random effects in the model.

The GCC is a generalized measure of association between variables. The association strength, which is called A, ranges from 0 (in case variables are independent) to 1 (in the case of perfect association between variables). A is the square of the correlation coefficient and can be considered as the proportion of variance in one variable explained by another variable or a number of variables. As the explained proportion of variance is one minus the unexplained proportion of variance, then the proportion of explained variance, R^2^, can be expressed as: 1-$\;\sigma_{error}^{2}/\sigma_{total}^{2}$, where $\sigma_{total}^{2}$ and $\;\sigma_{error}^{2}$ are average square deviations from a flat “null” model and a deterministic “alternative” model. Based on the assumption of normality of observations from least square regression, the deviation from a point on the regression line can be expressed as a probability density [9]. In a different way, the null model in GCC assumes that the variables are independent so the joint density for two variables *x* and *y* is the product of the marginal density estimation as *P*(*x*) *P*(*y*). The alternative model is considered as a mixture distribution of both dependent and independent components. It calculates a generalized R^2^ based on the probability density ratio of null and alternative models [9].

$$R^{2}=1-\prod_{i}{\left(\frac{P(xi,yi|null)}{P(xi,yi|alt)}\right)}^{2/n}$$ (1)

To calculate a p-value for assessing the significance of the test, i.e. to check the departure of observations from independence, it calculates a cross-validation likelihood for both the null and the alternative model. Equation (2) shows the calculation of the cross-validation likelihood statistics (*CVLRS*):

$$CVLRS=-2log\left(\frac{Lcv\left(Null\right)}{Lcv\left(Alt\right)}\right),$$ (2)

where *Lcv(Null)* and *Lcv(Alt)* are cross-validation likelihoods for the null and alternative models [9].

### Post-GWAS analysis

### Gene-based test and functional mapping in FUMA

Genomic risk loci were defined from the SNP-based GWAS results of the GCC, kinship and LME model analyses using functional mapping and annotation of genetic associations (FUMA) [42]. As part of post-GWAS analyses, a gene-based test was performed in MAGMA [43] integrated in FUMA. From GWAS summary statistic results, it tests the joint distribution of all SNP p-values. It maps SNPs to genes (window threshold = 10kb) based on their genomic location, using 1000 Genomes Phase3 as a reference, and LD within and between genes is determined. Furthermore, we investigated eQTL mapping, using the GTEx portal (https://gtexportal.org/home/), for the significant SNPs from the GCC-based GWAS to detect tissue-specific gene expression associated with the identified genetic.

### Gene-set enrichment analysis (GSEA)

To further investigate the biological function of the genes identified in the gene-based analysis from MAGMA, we used the top genes with p < 0.05 identified using the results of analyses with each of the three models in both Danish and Chinese samples as input for GSEA using KEGG pathways (N = 186). A hypergeometric test is used in GSEA (http://software.broadinstitute.org/gsea/index.jsp) to test the enrichment of genes against the KEGG pathways from MSigDB gene-sets. Results with FDR q-value < 0.05 are reported.

## Supplementary Material

Supplementary Figures

Supplementary Tables
