# Supplementary material for: Generalized correlation coefficient for genome-wide association analysis of cognitive ability in twins
Source: Aging (Albany NY). 2020 Nov 24;12(22):22457–94. doi: 10.18632/aging.104198 (PMC7746382; doi:10.18632/aging.104198)
Supplement: Supplementary Figures [file aging-12-104198-s001..pdf]

SUPPLEMENTARY FIGURES

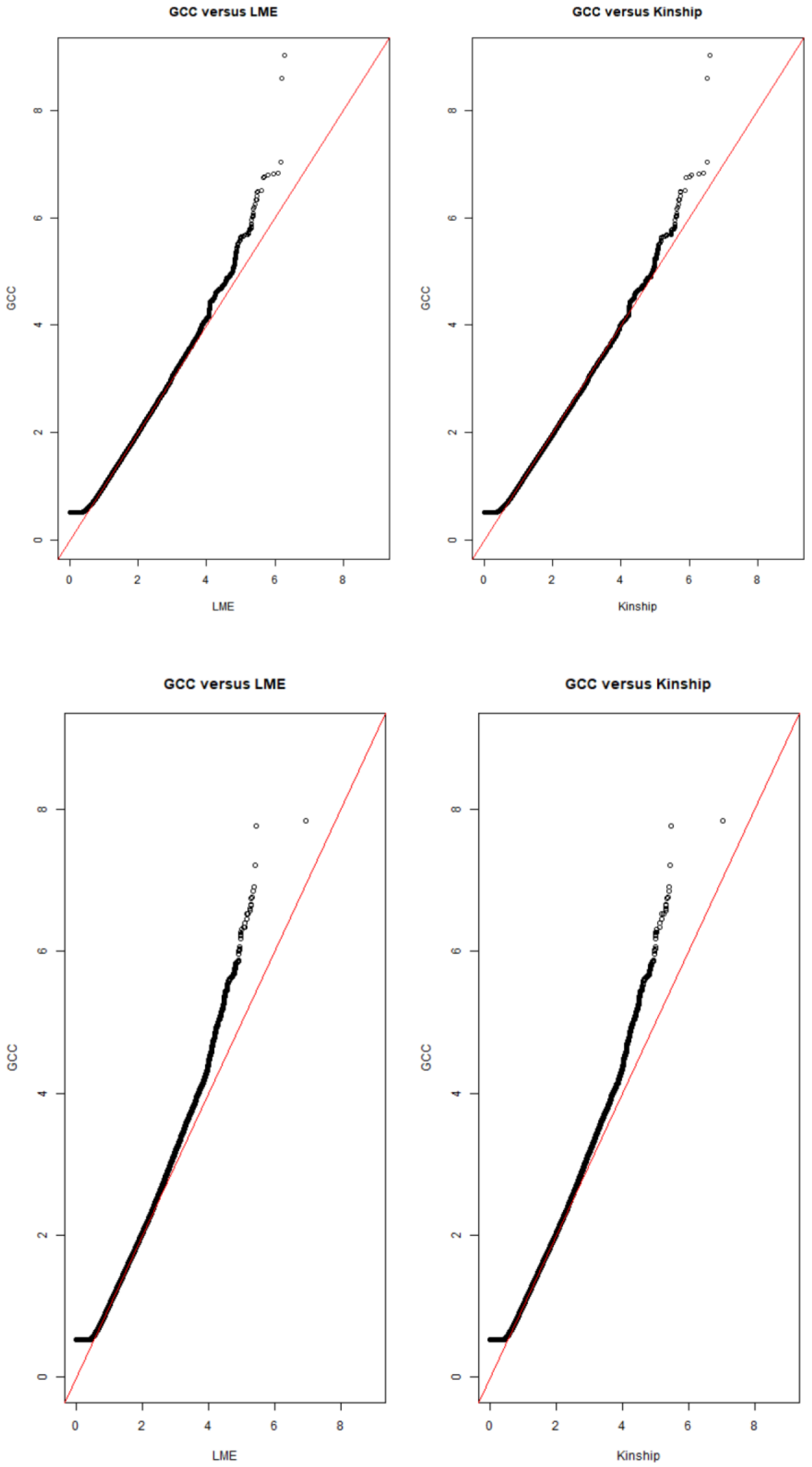

Supplementary Figure 1. QQ Plots comparing the GCC model to the kinship and LME models in Danish (upper plot) and Chinese (lower plot) data. The x-axis in both the left and right plots shows -log10 of p-values from GWAS using the LME and Kinship models, respectively. The y-axis in both plots shows -log10 of p-values from GWAS using the GCC model.

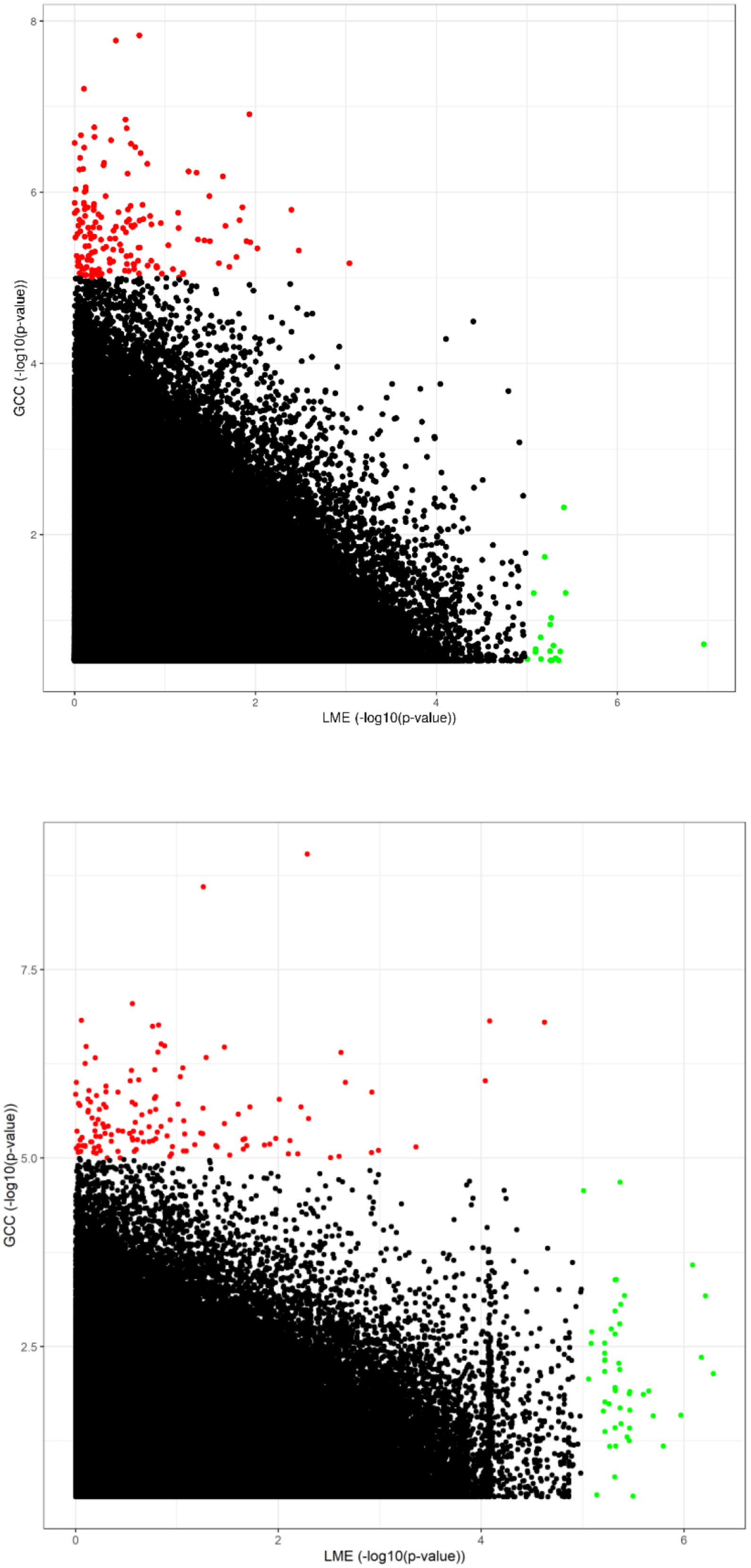

Supplementary Figure 2. Scatter plot comparing SNP p-values from the GCC GWAS to SNP p-values from the LME model GWAS in both Danish (upper plot) and Chinese (lower plot) data. The x-axis in both plots shows -log10 of SNP p-values from the LME GWAS. The y-axis in both plots shows -log10 SNP p-values from the GCC model GWAS.

A

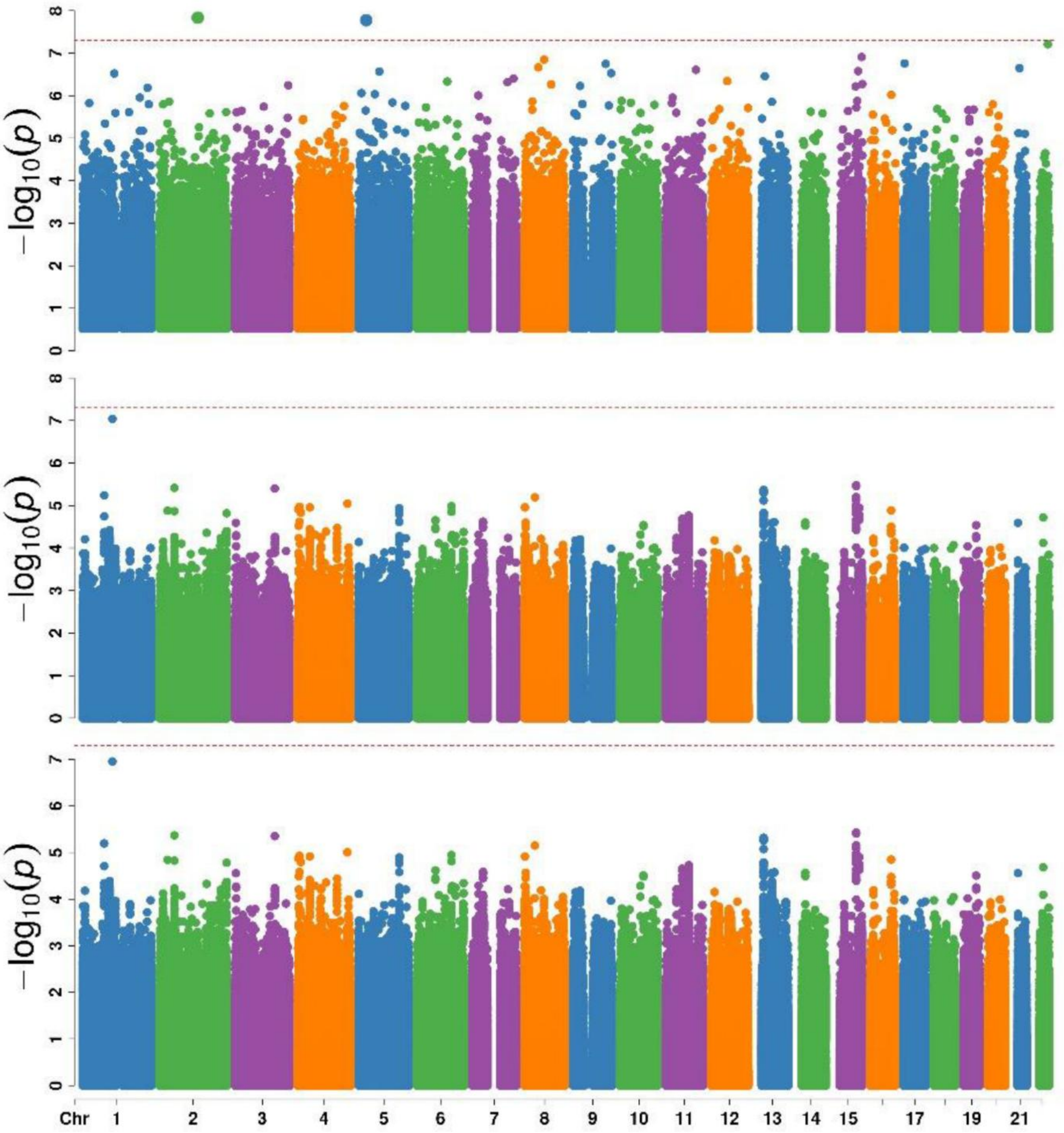

B

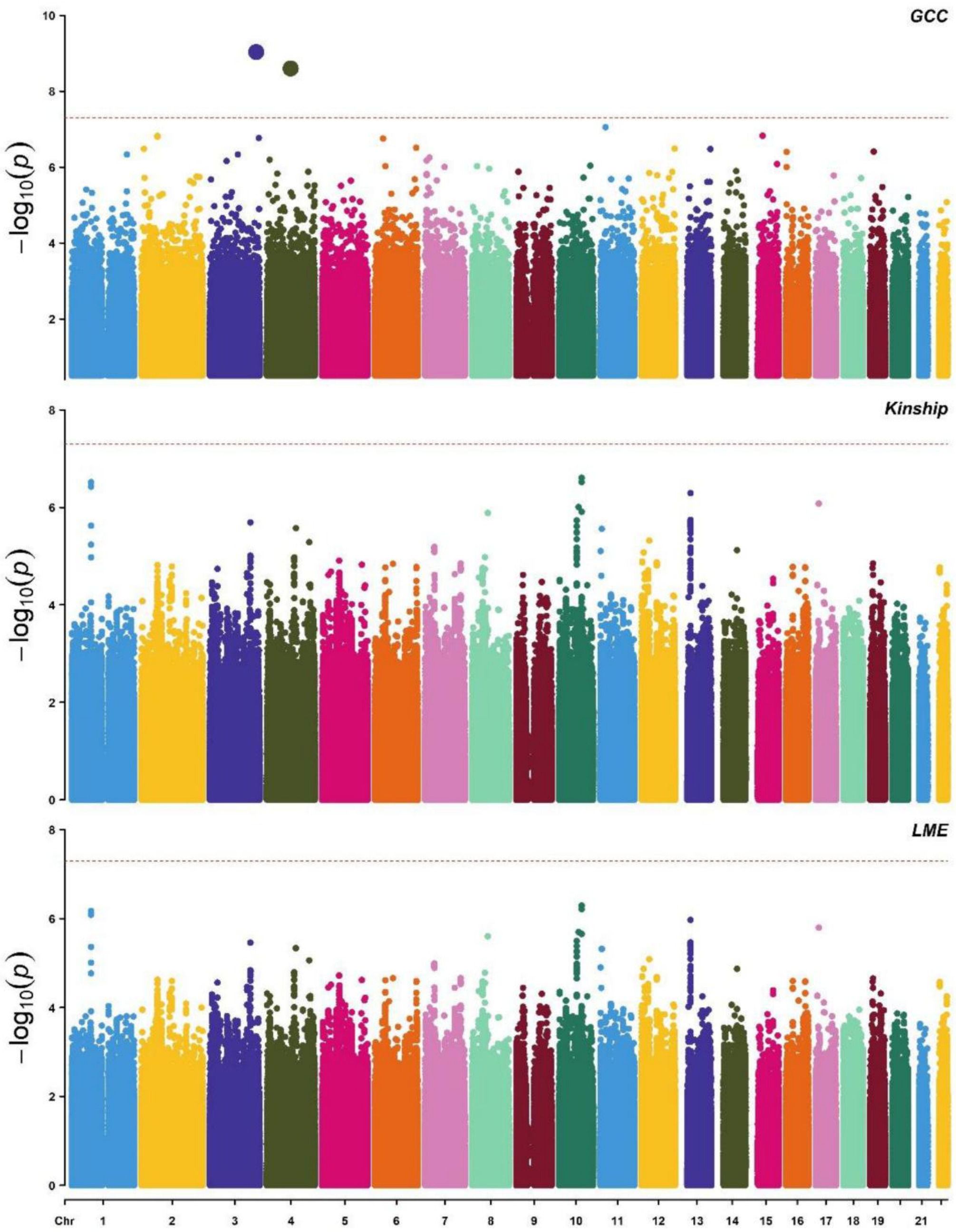

Supplementary Figure 3. Manhattan plots of the GCC, Kinship and LME GWAS results for the discovery data (**A**) and the replication data (**B**). The Manhattan plots in each plot from top to bottom are GCC GWAS, Kinship GWAS and LME GWAS, respectively.

A **Functional consequences of SNPs on genes**

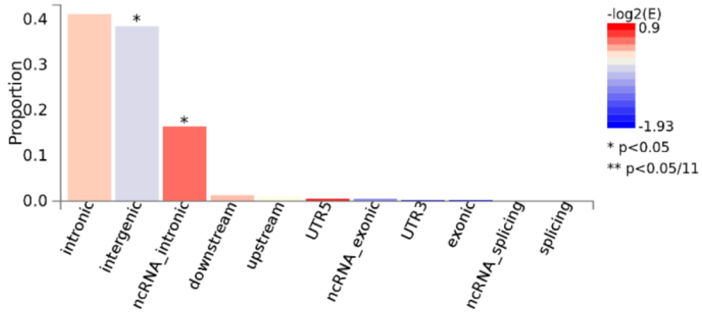

B **Summary of SNPs and mapped genes**

#Genomic risk loci	29
#Lead SNPs	29
#Ind. Sig. SNPs	29
#candidate SNPs	399
#candidate GWAS tagged SNPs	219
#mapped genes	862

C **Summary per genomic risk locus**

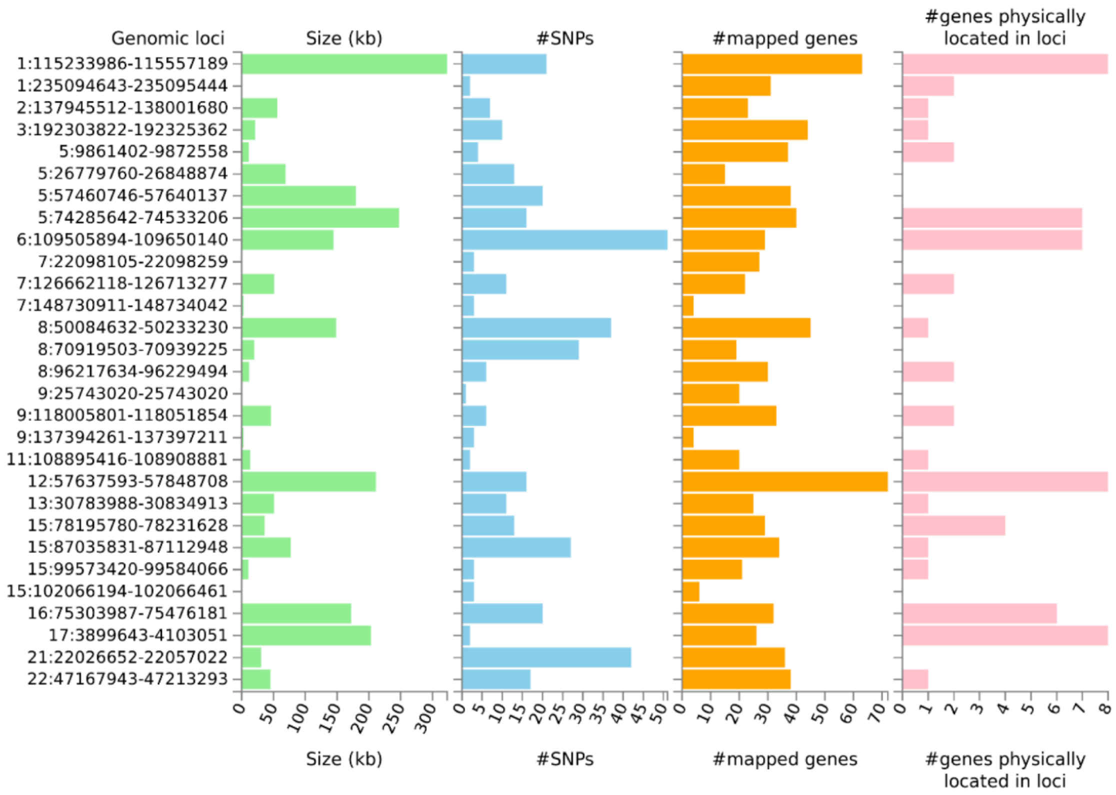

Supplementary Figure 4. Post-GWAS summary from FUMA of SNPs and mapped genes for the GCC GWAS in the Danish sample. (**A**) The histogram shows the proportion of SNPs that are in LD with independent significant SNPs, which have corresponding functional annotation assigned by ANNOVAR. Bars are colored log2 (enrichment) related to all SNPs in the reference panel. (**B**) The table shows summary of the number of SNPs and genes. (**C**) The histogram shows summary of results per genomic risk loci and genomic loci which could contain more than one independent lead SNPs.

A **Functional consequences of SNPs on genes**

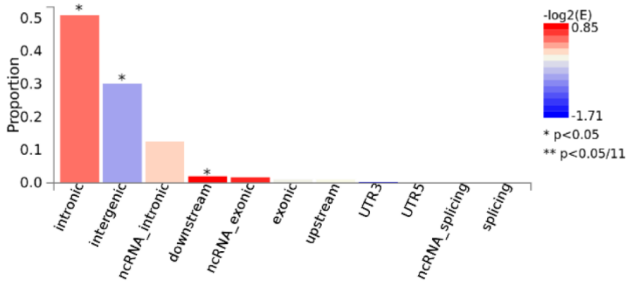

B **summary of SNPs and mapped genes**

#Genomic risk loci	24
#Lead SNPs	24
#Ind. Sig. SNPs	24
#candidate SNPs	705
#candidate GWAS tagged SNPs	501
#mapped genes	618

C **Summary per genomic risk loci**

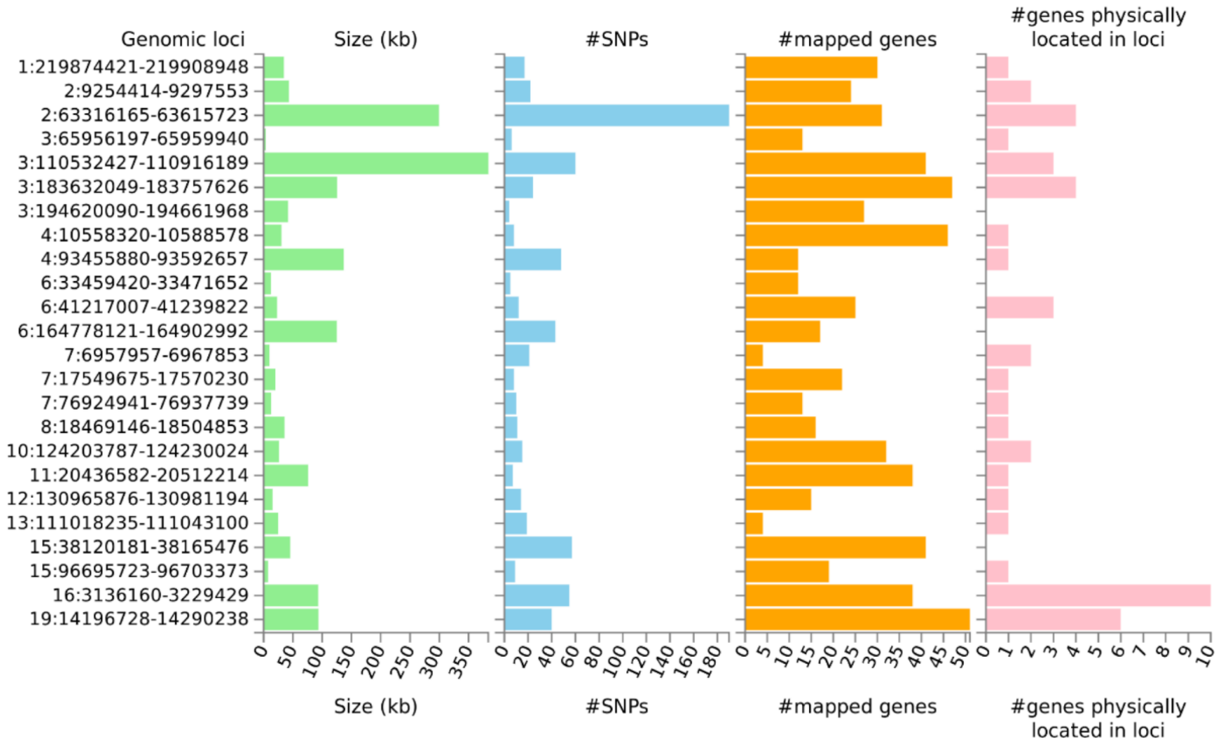

Supplementary Figure 5. Post-GWAS summary from FUMA of SNPs and mapped genes for the GCC GWAS in the Chinese sample. (**A**) The histogram shows the proportion of SNPs that are in LD with independent significant SNPs which have corresponding functional annotation assigned by ANNOVAR. Bars are colored log2(enrichment) related to all SNPs in the reference panel. (**B**) The table shows summary of the number of SNPs and genes. (**C**) The histogram shows summary of results per genomic risk loci and genomic loci which could contain more than one independent lead SNPs.

A **Functional consequences of SNPs on genes**

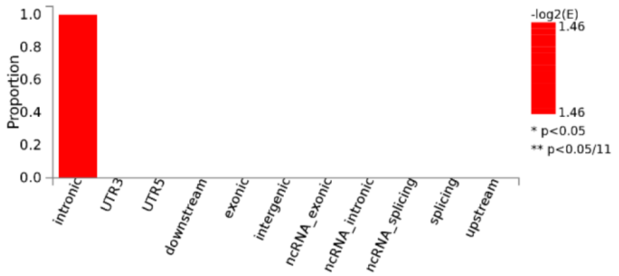

B **Summary of SNPs and mapped genes**

#Genomic risk loci	1
#Lead SNPs	1
#Ind. Sig. SNPs	1
#candidate SNPs	1
#candidate GWAS tagged SNPs	1
#mapped genes	14

C **Summary per genomic risk loci**

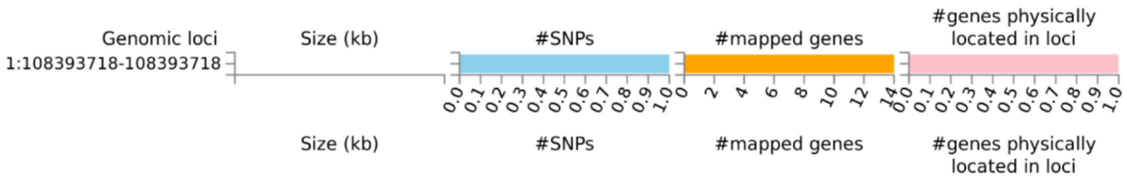

Supplementary Figure 6. Post-GWAS summary from FUMA of SNPs and mapped genes for the Kinship and LME GWAS in the Danish sample. (**A**) The histogram shows the proportion of SNPs that are in LD with independent significant SNPs, which have corresponding functional annotation assigned by ANNOVAR. Bars are colored log2(enrichment) related to all SNPs in the reference panel. (**B**) The table shows summary of the number of SNPs and genes. (**C**) The histogram shows summary of results per genomic risk loci and genomic loci which could contain more than one independent lead SNPs.

A Functional consequences of SNPs on genes genes

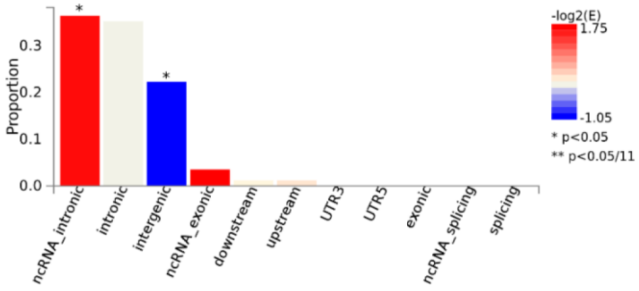

B Summary of SNPs and mapped

#Genomic risk loci	5
#Lead SNPs	5
#Ind. Sig. SNPs	5
#candidate SNPs	85
#candidate GWAS tagged SNPs	77
#mapped genes	161

C Summary per genomic risk locus

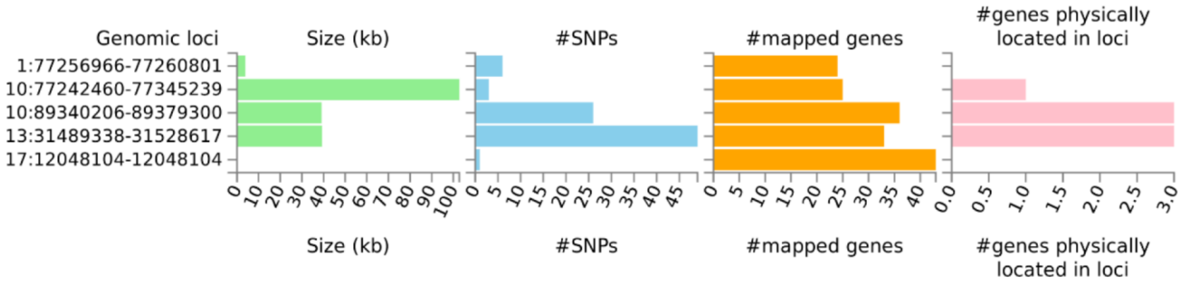

Supplementary Figure 7. Post-GWAS summary from FUMA of SNPs and mapped genes for the Kinship GWAS in the Chinese sample. (**A**) The histogram shows the proportion of SNPs that are in LD with independent significant SNPs, which have corresponding functional annotation assigned by ANNOVAR. Bars are colored log2(enrichment) related to all SNPs in the reference panel. (**B**) The table shows summary of the number of SNPs and genes. (**C**) The histogram shows summary of results per genomic risk loci and genomic loci which could contain more than one independent lead SNPs.

A Functional consequences of SNPs on genes
genes

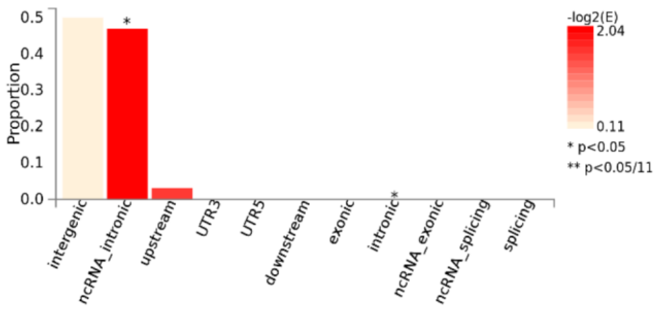

B Summary of SNPs and mapped

#Genomic risk loci	2
#Lead SNPs	2
#Ind. Sig. SNPs	2
#candidate SNPs	32
#candidate GWAS tagged SNPs	30
#mapped genes	60

C Summary per genomic risk locus

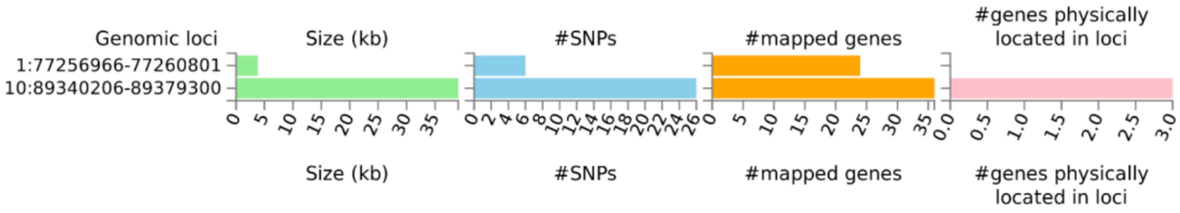

Supplementary Figure 8. Post-GWAS summary from FUMA of SNPs and mapped genes for the LME GWAS in the Danish sample. (**A**) The histogram shows the proportion of SNPs that are in LD with independent significant SNPs, which have corresponding functional annotation assigned by ANNOVAR. Bars are colored log2 (enrichment) related to all SNPs in the reference panel. (**B**) The table shows summary of the number of SNPs and genes. (**C**) The histogram shows summary of results per genomic risk loci and genomic loci which could contain more than one independent lead SNPs.
