# Supplementary material for: Generalized correlation coefficient for genome-wide association analysis of cognitive ability in twins
Source: Aging (Albany NY). 2020 Nov 24;12(22):22457–94. doi: 10.18632/aging.104198 (PMC7746382; doi:10.18632/aging.104198)
Supplement: Supplementary Tables [file aging-12-104198-s002..pdf]

SUPPLEMENTARY TABLES

Supplementary Table 1. Summary statistics for the top 30 SNPs identified in the GWAS of cognitive function in Danish samples using the GCC model. A and rawA are association scores from the matie R package.

Chr:Position	SNP ID	A1	A2	MAF*	INFO*	A	rawA	P-value
2:137996342	rs71419535	C	T	0.248	0.994	0.0920	0.0397	1.47e-08
5:26779974	rs905838	G	A	0.401	0.967	0.0916	0.0394	1.69e-08
22:47168581	rs6008973	A	G	0.453	0.963	0.0879	0.0368	6.19e-08
15:99584066	rs8023294	G	A	0.068	0.912	0.0858	0.0354	1.23e-07
8:70935439	rs56065347	A	G	0.168	0.992	0.0854	0.0352	1.42e-07
17:4103051	rs62064368	T	A	0.051	0.900	0.0848	0.0347	1.74e-07
9:118044633	rs1100324	G	A	0.500	0.990	0.0847	0.0347	1.79e-07
8:50093277	rs1509086	T	C	0.473	0.999	0.0841	0.0343	2.16e-07
21:22035477	rs2826435	A	C	0.161	0.951	0.0840	0.0342	2.25e-07
11:108908881	rs11604258	A	G	0.132	0.993	0.0837	0.0340	2.46e-07
15:87071809	rs4516199	G	A	0.478	0.939	0.0835	0.0339	2.66e-07
5:74310301	rs115636606	G	A	0.077	0.992	0.0834	0.0338	2.71e-07
9:137397211	rs7859114	T	C	0.146	0.653	0.0831	0.0337	2.97e-07
1:115369112	rs6689326	T	A	0.460	0.996	0.0831	0.0336	3.01e-07
13:30798807	rs861727	G	C	0.333	0.940	0.0826	0.0333	3.49e-07
7:148734042	rs4483075	T	C	0.105	0.820	0.0822	0.0331	3.96e-07
12:57835965	rs2943693	C	T	0.222	0.972	0.0818	0.0328	4.53e-07
6:109522751	rs1271693	T	C	0.298	0.997	0.0817	0.0327	4.66e-07
7:126707558	rs3808123	T	C	0.419	0.998	0.0816	0.0326	4.84e-07
15:102066194	rs2084151	G	C	0.452	0.994	0.0813	0.0324	5.33e-07
8:96229494	rs74306623	G	A	0.162	0.916	0.0812	0.0324	5.43e-07
3:192325362	rs7610664	T	C	0.158	0.900	0.0810	0.0323	5.71e-07
9:25743020	rs10491876	G	A	0.129	0.647	0.0809	0.0322	5.90e-07
15:78231397	rs12901519	A	G	0.280	0.889	0.0808	0.0322	6.06e-07
1:235095444	rs2802929	A	G	0.358	0.679	0.0806	0.0320	6.53e-07
5:9872558	rs2963897	A	G	0.339	0.650	0.0797	0.0314	8.71e-07
5:57640137	rs245295	A	T	0.094	0.990	0.0795	0.0313	9.24e-07
16:75377514	rs9939080	A	G	0.20	0.749	0.0793	0.0312	9.60e-07
7:22098259	rs1833111	G	A	0.3550	0.749	0.0792	0.0312	9.88e-07
1:207421048	rs7535029	A	G	0.320	0.996	0.0789	0.0309	1.11e-06

*MAF = Minor allele Frequency,
*INFO = Information metric score from the imputation.

Supplementary Table 2. Summary statistic for the top 30 SNPs from Kinship model in Danish GWAS.

Chr:Position	SNPID	A1	A2	MAF*	INFO*	BETA	SE	Z	P-value
1:108393718	rs72707744	G	A	0.1012	0.7436	-3.6972	0.6919	-5.34	9.11e-08
15:80099089	rs34903151	T	C	0.105	0.922	3.0052	0.6464	4.65	3.34e-06
15:80099202	rs59367695	G	A	0.106	0.925	2.9949	0.6456	4.64	3.50e-06
2:53701324	rs903227	C	T	0.179	0.959	-2.3474	0.508	-4.62	3.82e-06
3:143762041	rs4839678	G	A	0.1656	0.7519	-2.6011	0.5639	-4.61	3.98e-06
13:26639396	rs7491901	A	G	0.4421	0.9979	1.8016	0.392	4.6	4.30e-06
13:26642629	rs7321426	C	T	0.4415	0.9973	1.7984	0.3923	4.58	4.57e-06
13:26641424	rs9512064	C	G	0.4416	0.9977	1.7949	0.3922	4.58	4.72e-06
13:26640931	rs9553716	T	A	0.4418	0.9976	1.7931	0.3922	4.57	4.84e-06
13:26634144	rs9512063	T	C	0.4416	0.9984	1.7918	0.3923	4.57	4.94e-06
13:26638368	rs1853720	C	A	0.4415	0.9987	1.7913	0.3923	4.57	4.97e-06
13:26636704	rs7317113	C	T	0.4415	0.9981	1.7908	0.3923	4.56	5.00e-06
1:78623626	rs17391694	C	T	0.1320	1	2.532	0.5582	4.54	5.74e-06
15:80119888	rs16971397	A	G	0.117	0.969	2.7288	0.6043	4.52	6.31e-06
8:37669354	rs10089977	T	C	0.2318	0.6758	2.4104	0.5341	4.51	6.39e-06
15:80103298	rs66588428	C	G	0.1103	0.941	2.8405	0.6332	4.49	7.26e-06
15:80100919	rs7183155	A	T	0.1098	0.932	2.8574	0.6372	4.48	7.31e-06
13:26637596	rs4770916	C	T	0.442	1	1.7614	0.3936	4.48	7.62e-06
4:178681212	rs10222887	G	T	0.3944	0.9902	1.6841	0.3793	4.44	8.99e-06
15:80151536	rs12912711	G	A	0.112	0.981	2.7315	0.6165	4.43	9.41e-06
15:80123923	rs9888692	G	A	0.113	0.992	2.7027	0.6108	4.43	9.64e-06
15:80122034	rs71409256	G	A	0.113	0.993	2.701	0.6108	4.42	9.80e-06
15:80143118	rs8033289	G	A	0.112	0.986	2.7199	0.6156	4.42	9.96e-06
15:80131092	rs7174937	G	A	0.113	0.990	2.7092	0.6134	4.42	1.00e-05
15:80141806	rs36060920	C	G	0.112	0.9868	2.7178	0.6155	4.42	1.01e-05
15:80140678	rs36107316	C	T	0.113	0.987	2.7176	0.6157	4.41	1.01e-05
6:124643640	rs2626123	G	C	0.2500	0.8012	-2.1333	0.4836	-4.41	1.03e-05
4:6196861	rs11729302	C	G	0.1158	0.6010	-3.3278	0.7559	-4.4	1.07e-05
15:80137560	exm1181778	T	C	0.113	1	2.6806	0.6094	4.4	1.09e-05
8:1886823	rs747689	C	T	0.3534	0.7651	1.9831	0.45	4.4	1-10e-05

*MAF = Minor allele Frequency,
*INFO = Information metric score from the imputation.

Supplementary Table 3. Summary statistic for the top 30 SNPs from LME model in Danish GWAS.

Chr:Position	SNP ID	A1	A2	MAF*	INFO*	Coef	tval	SE	P-value
1:108393718	rs72707744	G	A	0.1012	0.7436	-3.6972	-5.3437	0.6919	1.10e-07
15:80099089	rs34903151	T	C	0.105	0.922	3.0052	4.649	0.6464	3.73e-06
15:80099202	rs59367695	G	A	0.106	0.925	2.9949	4.6391	0.6456	3.91e-06
2:53701324	rs903227	C	T	0.179	0.959	-2.3474	-4.6206	0.508	4.27e-06
3:143762041	rs4839678	G	A	0.1656	0.7519	-2.6011	-4.6124	0.5639	4.43e-06
13:26639396	rs7491901	A	G	0.4421	0.9979	1.8016	4.5964	0.392	4.78e-06
13:26642629	rs7321426	C	T	0.4415	0.9973	1.7984	4.5836	0.3923	5.08e-06
13:26641424	rs9512064	C	G	0.4416	0.9977	1.7949	4.5767	0.3922	5.25e-06
13:26640931	rs9553716	T	A	0.4418	0.9976	1.7931	4.5716	0.3922	5.37e-06
13:26634144	rs9512063	T	C	0.4416	0.9984	1.7918	4.5675	0.3923	5.48e-06
13:26638368	rs1853720	C	A	0.4415	0.9987	1.7913	4.5659	0.3923	5.52e-06
13:26636704	rs7317113	C	T	0.4415	0.9981	1.7908	4.5649	0.3923	5.54e-06
1:78623626	rs17391694	C	T	0.1320	1	2.532	4.5357	0.5582	6.35e-06
15:80119888	rs16971397	A	G	0.117	0.969	2.7288	4.5158	0.6043	6.97e-06
8:37669354	rs10089977	T	C	0.2318	0.6758	2.4104	4.5131	0.5341	7.04e-06
15:80103298	rs66588428	C	G	0.1103	0.941	2.8405	4.4859	0.6332	8.00e-06
15:80100919	rs7183155	A	T	0.1098	0.932	2.8574	4.4845	0.6372	8.05e-06
13:26637596	rs4770916	C	T	0.442	1	1.7614	4.4756	0.3936	8.39e-06
4:178681212	rs10222887	C	T	0.3944	0.9902	1.6841	4.4401	0.3793	9.87e-06
15:80151536	rs12912711	G	A	0.112	0.981	2.7315	4.4303	0.6166	1.03e-05
15:80123923	rs9888692	G	A	0.113	0.992	2.7027	4.4251	0.6108	1.06e-05
15:80122034	rs71409256	G	A	0.113	0.993	2.7009	4.4216	0.6108	1.07e-05
15:80143118	rs8033289	G	A	0.112	0.986	2.7198	4.4179	0.6156	1.09e-05
15:80131092	rs7174937	G	A	0.113	0.990	2.7092	4.417	0.6134	1.10e-05
15:80141806	rs36060920	C	G	0.112	0.9868	2.7178	4.4159	0.6155	1.10e-05
15:80140678	rs36107316	C	T	0.113	0.987	2.7176	4.4142	0.6157	1.11e-05
6:124643640	rs2626123	G	C	0.2500	0.8012	-2.1333	-4.4114	0.4836	1.13e-05
4:6196861	rs11729302	C	G	0.1158	0.6010	-3.3278	-4.4021	0.756	1.17e-05
15:80137560	exm1181778	T	C	0.113	1	2.6806	4.3991	0.6094	1.19e-05
4:43193184	rs62299190	T	C	0.1139	0.8934	2.7868	4.3958	0.634	1.21e-05

*MAF = Minor allele Frequency,
*INFO = Information metric score.

Supplementary Table 4. Summary statistic for the top 30 SNPs from GCC in Chinese GWAS. A and rawA are association scores from matie R package.

Chr: Position	SNP ID	A1	A2	MAF*	INFO*	A	rawA	P-value
3:183681056	rs2292999	T	G	0.15108	1	0.2501	0.1697	9.27e-10
4:93505196	rs17019635	A	G	0.4479	0.99973	0.2436	0.1628	2.50e-09
11:20440691	rs12362967	A	G	0.2532	0.99503	0.2188	0.1373	8.95e-08
15:38153188	rs1502398	A	T	0.23565	0.99978	0.2151	0.1336	1.49e-07
2:63447200	rs13417792	G	T	0.05039	0.99921	0.2150	0.1335	1.51e-07
2:63439916	rs62177776	C	A	0.0543	0.91907	0.2146	0.1332	1.58e-07
3:194620090	rs61253693	G	A	0.23285	0.99638	0.2140	0.1326	1.71e-07
6:33459420	rs11757081	A	T	0.08714	0.98874	0.2138	0.1323	1.78e-07
6:164790026	rs10945984	C	T	0.45905	0.97115	0.2097	0.1282	3.07e-07
12:130968399	rs59068957	G	A	0.15953	0.94953	0.2093	0.1278	3.25e-07
2:9278695	rs7590864	T	A	0.10432	0.99966	0.2091	0.1276	3.31e-07
13:111036022	rs9515200	G	C	0.44155	0.9936	0.2090	0.1275	3.36e-07
19:14241153	rs8112885	A	G	0.10304	0.99419	0.2079	0.1264	3.91e-07
16:3181996	rs11649450	G	A	0.1781	0.90386	0.2078	0.1263	3.95e-07
3:110692754	rs1462795	T	C	0.46763	1	0.2066	0.1251	4.64e-07
1:219903142	rs4120823	C	T	0.31726	0.98683	0.2066	0.1251	4.65e-07
7:17554180	rs7808662	G	A	0.25336	0.9957	0.2052	0.1237	5.54e-07
4:10558320	rs17382947	A	G	0.09376	0.98675	0.2042	0.1227	6.38e-07
7:6964606	rs58404784	C	T	0.11034	0.76589	0.2037	0.1222	6.74e-07
3:65958585	rs17074200	A	T	0.266	0.99904	0.2036	0.1221	6.87e-07
15:96698614	rs727895	T	G	0.47377	0.99825	0.2021	0.1207	8.30e-07
10:124213046	rs61871747	C	T	0.45393	0.9945	0.2014	0.1199	9.13e-07
6:41222926	rs1872245	C	T	0.34691	0.99671	0.2012	0.1197	9.39e-07
8:18469146	rs77159425	A	G	0.11372	0.98384	0.2011	0.1197	9.43e-07
7:76927290	rs76326531	G	T	0.19772	0.98887	0.2008	0.1193	9.88e-07
16:3181963	rs1053871	T	C	0.1788	0.9017	0.2008	0.1193	9.91e-07
8:67171047	rs74193350	T	C	0.12023	0.9973	0.1999	0.1184	1.11e-06
14:69078998	rs7153168	C	T	0.16727	0.99998	0.1988	0.1174	1.27e-06
9:8011093	rs4740923	G	A	0.05204	0.91471	0.1985	0.1170	1.33e-06
4:164850409	rs55899995	A	G	0.05412	0.99699	0.1985	0.1170	1.33e-06

*MAF = Minor allele Frequency,
*INFO = Information metric score.

Supplementary Table 5. Summary statistic for the top 30 SNPs from Kinship model in Chinese GWAS.

Chr: Position	SNP ID	A1	A2	MAF*	INFO*	Beta	SE	z	P-value
10:89340206	rs10749553	A	G	0.37741	0.93624	-70.5629	13.6776	-5.16	2.48e-07
1:77259107	rs11577464	A	G	0.18385	0.9976	-87.3978	17.0651	-5.12	3.03e-07
10:89341315	rs10749555	A	C	0.37628	0.94323	-69.8189	13.6345	-5.12	3.04e-07
1:77260333	rs61783213	C	T	0.18491	0.96408	-86.9847	17.1274	-5.08	3.80e-07
13:31508724	rs1331848	A	G	0.44958	0.99976	64.3429	12.8125	5.02	5.12e-07
17:12048104	rs12944210	A	C	0.07151	0.98875	-126.6781	25.7148	-4.93	8.38e-07
10:77242460	rs117082223	G	A	0.0603	0.63757	169.5016	34.6389	4.89	9.91e-07
10:89340727	rs10887729	A	T	0.46505	0.93468	-66.7767	13.7705	-4.85	1.24e-06
8:61573242	rs56274310	C	G	0.15584	0.93985	87.7518	18.1382	4.84	1.31e-06
13:31513622	rs7319162	T	C	0.44242	0.9997	61.1840	12.8201	4.77	1.82e-06
13:31513145	rs4943681	G	A	0.44245	1	61.1749	12.8188	4.77	1.82e-06
13:31512404	rs2872194	A	G	0.44248	0.99981	61.1677	12.8205	4.77	1.83e-06
13:31512486	rs2313006	T	C	0.44256	0.99939	61.1571	12.8222	4.77	1.85e-06
10:70280398	rs78573780	A	T	0.31538	0.96562	-70.4836	14.7817	-4.77	1.86e-06
13:31509070	rs7321378	A	T	0.44332	0.9977	60.8599	12.7927	4.76	1.96e-06
3:161523263	rs1436738	G	A	0.06914	0.96809	117.1424	24.6759	4.75	2.06e-06
13:31509870	rs7984339	G	A	0.44357	0.99829	-60.9480	12.8439	-4.75	2.08e-06
13:31507727	rs1002036	G	T	0.43885	0.99999	-62.8348	13.2757	-4.73	2.21e-06
13:31528505	rs59815783	C	T	0.44042	0.96004	61.7178	13.0697	4.72	2.33e-06
13:31514975	rs61947599	A	G	0.44402	0.99701	60.7065	12.8565	4.72	2.34e-06
13:31513931	rs2225505	T	A	0.44419	0.99913	60.6492	12.8448	4.72	2.34e-06
13:31513425	rs7325107	A	G	0.44424	1	60.6235	12.8406	4.72	2.34e-06
1:77257353	rs4949747	A	T	0.19922	0.96797	-79.0279	16.7488	-4.72	2.38e-06
10:70365455	rs73276500	A	C	0.32545	0.95417	-66.5443	14.1210	-4.71	2.45e-06
13:31526533	rs12864826	T	C	0.42596	0.99859	61.1288	12.9912	4.71	2.53e-06
13:31522359	rs4943792	C	T	0.42601	0.9986	61.1191	12.9917	4.7	2.55e-06
13:31515563	rs28582187	T	A	0.42624	0.99983	61.0835	12.9876	4.7	2.56e-06
13:31509744	rs4941921	G	A	0.42626	0.99999	61.0794	12.9875	4.7	2.56e-06
13:31515301	rs61580577	G	A	0.42626	1	61.0794	12.9875	4.7	2.56e-06
13:31517404	rs12870193	G	A	0.42617	0.99911	61.0942	12.9908	4.7	2.57e-06

*MAF = Minor allele Frequency,
*INFO = Information metric score.

Supplementary Table 6. Summary statistic for the top 30 SNPs from LME model in Chinese GWAS.

Chr: Position	SNP ID	A1	A2	MAF*	INFO*	Coef	SE	t-value	P-value
10:89340206	rs10749553	A	G	0.37741	0.93624	-70.6351	13.7296	-5.1447	5.11e-07
10:89341315	rs10749555	A	C	0.37628	0.94323	-69.8916	13.6862	-5.1067	6.14e-07
1:77259107	rs11577464	A	G	0.18385	0.9976	-87.2301	17.1337	-5.0911	6.73e-07
1:77260333	rs61783213	C	T	0.18491	0.96408	-86.8156	17.1957	-5.0487	8.23e-07
13:31508724	rs1331848	A	G	0.44958	0.99976	64.2952	12.8663	4.9972	1.07e-06
17:12048104	rs12944210	A	C	0.07151	0.98875	-126.7369	25.8196	-4.9085	1.60e-06
10:77242460	rs117082223	G	A	0.0603	0.63757	169.3622	34.7934	4.8677	2.02e-06
10:89340727	rs10887729	A	T	0.46505	0.93468	-66.8073	13.8211	-4.8337	2.23e-06
8:61573242	rs56274310	C	G	0.15584	0.93985	87.5579	18.2082	4.8087	2.52e-06
10:70280398	rs78573780	A	T	0.31538	0.96562	-70.5498	14.8339	-4.7560	3.19e-06
13:31513622	rs7319162	T	C	0.44242	0.9997	61.1277	12.8743	4.7480	3.41e-06
13:31513145	rs4943681	G	A	0.44245	1	61.1187	12.8730	4.7478	3.41e-06
13:31512404	rs2872194	A	G	0.44248	0.99981	61.1114	12.8747	4.7466	3.43e-06
13:31512486	rs2313006	T	C	0.44256	0.99939	61.1009	12.8763	4.7452	3.45e-06
3:161523263	rs1436738	G	A	0.06914	0.96809	117.3295	24.7618	4.7383	3.46e-06
13:31509070	rs7321378	A	T	0.44332	0.9977	60.7993	12.8465	4.7328	3.65e-06
13:31509870	rs7984339	G	A	0.44357	0.99829	-60.8889	12.8979	-4.7208	3.85e-06
10:70365455	rs73276500	A	C	0.32545	0.95417	-66.5908	14.1752	-4.6977	4.18e-06
13:31507727	rs1002036	G	T	0.43885	0.99999	-62.6854	13.3314	-4.7021	4.19e-06
13:31528505	rs59815783	C	T	0.44042	0.96004	61.6639	13.1247	4.6983	4.27e-06
13:31514975	rs61947599	A	G	0.44402	0.99701	60.6452	12.9104	4.6974	4.28e-06
13:31513931	rs2225505	T	A	0.44419	0.99913	60.5882	12.8987	4.6972	4.28e-06
13:31513425	rs7325107	A	G	0.44424	1	60.5626	12.8944	4.6968	4.29e-06
1:77257353	rs4949747	A	T	0.19922	0.96797	-78.8652	16.8166	-4.6897	4.38e-06
4:115490688	rs112169253	A	C	0.05673	0.78317	-143.0388	30.5930	-4.6755	4.64e-06
13:31526533	rs12864826	T	C	0.42596	0.99859	61.0632	13.0485	4.6797	4.72e-06
13:31522359	rs4943792	C	T	0.42601	0.9986	61.0534	13.0491	4.6788	4.74e-06
13:31515563	rs28582187	T	A	0.42624	0.99983	61.0180	13.0449	4.6775	4.77e-06
13:31509744	rs4941921	G	A	0.42626	0.99999	61.0140	13.0449	4.6772	4.77e-06
13:31515301	rs61580577	G	A	0.42626	1	61.0140	13.0449	4.6772	4.77e-06

*MAF = Minor allele Frequency,
*INFO = Information metric score

Supplementary Table 7. Top 20 genes from the MAGMA gene-based analysis of the Danish GCC GWAS results.

SYMBOL	CHR	START	STOP	NSNPS	NPARAM	N	ZSTAT	P-value	FDR
CSMD1	8	2782875	4862494	12973	488	1172	4.8682	5.63e-07	0.011
PTPRD	9	8304246	10622723	7726	379	1172	4.148	1.68e-05	0.16
RBFOX1	16	6059095	7773340	7791	369	1172	4.0358	2.72e-05	0.17
CDH13	16	82650408	83840204	5249	284	1172	3.7244	9.79e-05	0.26
WWOX	16	78123310	79256564	5054	278	1172	3.5371	0.000202	0.26
CNTN4	3	2130497	3109645	3493	256	1172	3.4814	0.000249	0.26
FHIT	3	59725036	61247133	4957	279	1172	3.3964	0.000341	0.26
DPP6	7	153574182	154695995	3037	244	1172	3.3432	0.000414	0.26
TMEM132D	12	129546270	130398211	2565	223	1172	3.3348	0.000427	0.26
NPAS3	14	33394139	34283382	1963	214	1172	3.2302	0.000618	0.26
CDH4	20	59817482	60525673	2223	187	1172	3.2198	0.000641	0.26
MACROD2	20	13966015	16043842	4335	233	1172	3.1694	0.000764	0.26
OPCML	11	132274871	133412414	2616	209	1172	3.1024	0.00096	0.26
NTM	11	131230373	132216716	2400	186	1172	3.0947	0.000985	0.26
DLGAP1	18	3486030	4465335	2480	224	1172	3.0724	0.00106	0.26
KAZN	1	14915200	15454539	1511	143	1172	3.0629	0.0011	0.26
CTNNA2	2	79402357	80885905	3473	215	1172	3.03	0.00122	0.26
CNTNAP2	7	145803453	148128090	6261	209	1172	3.008	0.00131	0.26
SORCS2	4	7184265	7754554	2406	210	1172	3.0043	0.00133	0.26
ASIC2	17	31330105	32511983	3092	217	1172	2.9859	0.00141	0.26

Supplementary Table 8. Top 20 genes from GCC model in Chinese data from MAGMA.

SYMBOL	CHR	START	STOP	NSNPS	NPARAM	N	ZSTAT	P-value	FDR
CSMD1	8	2782875	4862494	12560	474	278	4.1834	1.44e-05	0.27
PTPRD	9	8304246	10622723	7351	367	278	3.778	7.91e-05	0.29
RBFOX1	16	6059095	7773340	7664	395	278	3.6857	0.000114	0.29
NPAS3	14	33394139	34283382	1986	220	278	3.6352	0.000139	0.29
WWOX	16	78123310	79256564	5118	317	278	3.3459	0.00041	0.29
CDH13	16	82650408	83840204	4127	267	278	3.2772	0.000524	0.29
CTNNA2	2	79402357	80885905	3820	267	278	3.1567	0.000798	0.29
KSR2	12	117880817	118416788	1435	158	278	3.1535	0.000807	0.29
DLGAP1	18	3486030	4465335	2422	226	278	3.1423	0.000838	0.29
DAB1	1	57450451	59022406	3461	211	278	3.142	0.000839	0.29
FRMD4A	10	13675706	14514141	2954	215	278	3.1174	0.000912	0.29
TMEM132D	12	129546270	130398211	2659	238	278	3.1167	0.000915	0.29
SORCS2	4	7184265	7754554	2084	206	278	3.1155	0.000918	0.29
MACROD2	20	13966015	16043842	4120	248	278	3.1149	0.00092	0.29
NELL1	11	20681117	21607227	2475	198	278	3.0328	0.00121	0.29
RYR2	1	237195505	238007288	2183	165	278	3.0075	0.00132	0.29
TMEM132C	12	128741948	129202460	1095	137	278	2.8947	0.0019	0.29
TEX26	13	31496840	31559639	77	7	278	2.8887	0.00193	0.29
LDLRAD4	18	13207497	13662754	985	114	278	2.8763	0.00201	0.29
MAGI2	7	77636393	79092890	4052	224	278	2.872	0.00204	0.29

Supplementary Table 9. The 49 significant (FDR< 0.05) KEGG pathways identified in the GSEA overrepresentation analysis based on genes found to have a p-value <0.05 in the gene-based analysis of GCC GWAS results in the Danish discovery sample.

Gene set names[# Genes (K)]	Description	#genes in overlap(K)	P-value	FDR q-value
KEGG_PATHWAYS_IN_CANCER [325]	Pathways in cancer	37	1.08 e^{-12}	2.01 e^{-10}
KEGG_AXON_GUIDANCE [129]	Axon guidance	21	1.16 e^{-10}	1.08 e^{-8}
KEGG_ARRHYTHMOGENIC_RIGHT_VENTRICULAR_ AR_CARDIOMYOPATHY_ARVC [74]	Arrhythmogenic right ventricular cardiomyopathy (ARVC)	15	2.27 e^{-9}	1.41 e^{-7}
KEGG_VASCULAR_SMOOTH_MUSCLE_CONTRACTIO TION [115]	Vascular smooth muscle contraction	18	4.67 e^{-9}	2.17 e^{-7}
KEGG_CALCIUM_SIGNALING_PATHWAY [177]	Calcium signaling pathway	22	8.07 e^{-9}	3 e^{-7}
KEGG_FOCAL_ADHESION [199]	Focal adhesion	23	1.51 e^{-8}	4.67 e^{-7}
KEGG_MAPK_SIGNALING_PATHWAY [267]	MAPK signaling pathway	25	2.35 e^{-7}	6.26 e^{-6}
KEGG_HYPERTROPHIC_CARDIOMYOPATHY_HCM [83]	Hypertrophic cardiomyopathy (HCM)	13	6.33 e^{-7}	1.47 e^{-5}
KEGG_WNT_SIGNALING_PATHWAY [150]	Wnt signaling pathway	17	1.46 e^{-6}	3.02 e^{-5}
KEGG_DILATED_CARDIOMYOPATHY [90]	Dilated cardiomyopathy	13	1.64 e^{-6}	3.04 e^{-5}
KEGG_LONG_TERM_DEPRESSION [70]	Long-term depression	11	4.43 e^{-6}	7.5 e^{-5}
KEGG_GNRH_SIGNALING_PATHWAY [101]	GnRH signaling pathway	13	6.09 e^{-6}	9.44 e^{-5}
KEGG_ADHERENS_JUNCTION [73]	Adherens junction	11	6.75 e^{-6}	9.65 e^{-5}
KEGG_O_GLYCAN_BIOSYNTHESIS [30]	O-Glycan biosynthesis	7	1.71 e^{-5}	2.27 e^{-4}
KEGG_BASAL_CELL_CARCINOMA [55]	Basal cell carcinoma	9	2.34 e^{-5}	2.9 e^{-4}
KEGG_HEDGEHOG_SIGNALING_PATHWAY [56]	Hedgehog signaling pathway	9	2.72 e^{-5}	2.94 e^{-4}
KEGG_LEUKOCYTE_TRANSENDOTHELIAL_MIGRAT RATION [116]	Leukocyte transendothelial migration	13	2.77 e^{-5}	2.94 e^{-4}
KEGG_LONG_TERM_POTENTIATION [70]	Long-term potentiation	10	2.84 e^{-5}	2.94 e^{-4}
KEGG_ERBB_SIGNALING_PATHWAY [87]	ErbB signaling pathway	11	3.7 e^{-5}	3.62 e^{-4}
KEGG_REGULATION_OF_ACTIN_CYTOSKELETON [213]	Regulation of actin cytoskeleton	18	4.39 e^{-5}	4.09 e^{-4}
KEGG_GAP_JUNCTION [90]	Gap junction	11	5.08 e^{-5}	4.5 e^{-4}
KEGG_PHOSPHATIDYLINOSITOL_SIGNALING_SY _SYSTEM [76]	Phosphatidylinositol signaling system	10	5.87 e^{-5}	4.96 e^{-4}
KEGG_FC_EPSILON_RI_SIGNALING_PATHWAY [79]	Fc epsilon RI signaling pathway	10	8.2 e^{-5}	6.63 e^{-4}
KEGG_CELL_ADHESION_MOLECULES_CAMS [133]	Cell adhesion molecules (CAMs)	13	1.16 e^{-4}	8.96 e^{-4}
KEGG_NON_SMALL_CELL_LUNG_CANCER [54]	Non-small cell lung cancer	8	1.38 e^{-4}	9.89 e^{-4}
KEGG_ECM_RECEPTOR_INTERACTION [84]	ECM-receptor interaction	10	1.38 e^{-4}	9.89 e^{-4}
KEGG_TYPE_II_DIABETES_MELLITUS [47]	Type II diabetes mellitus	7	3.47 e^{-4}	2.39 e^{-3}
KEGG_FC_GAMMA_R_MEDIATED_PHAGOCYTOSIS [96]	Fc gamma R-mediated phagocytosis	10	4.16 e^{-4}	2.76 e^{-3}
KEGG_INSULIN_SIGNALING_PATHWAY [137]	Insulin signaling pathway	12	5.73 e^{-4}	3.68 e^{-3}
KEGG_MELANOGENESIS [101]	Melanogenesis	10	6.24 e^{-4}	3.87 e^{-3}
KEGG_SMALL_CELL_LUNG_CANCER [84]	Small cell lung cancer	9	6.5 e^{-4}	3.9 e^{-3}
KEGG_ALDOSTERONE_REGULATED_SODIUM_REAB EABSORPTION [42]	Aldosterone-regulated sodium reabsorption	6	1.15 e^{-3}	6.69 e^{-3}
KEGG_B_CELL_RECEPTOR_SIGNALING_PATHWAY [75]	B cell receptor signaling pathway	8	1.32 e^{-3}	7.44 e^{-3}
KEGG_CARDIAC_MUSCLE_CONTRACTION [78]	Cardiac muscle contraction	8	1.7 e^{-3}	9.31 e^{-3}
KEGG_PURINE_METABOLISM [159]	Purine metabolism	12	2.09 e^{-3}	1.08 e^{-2}
KEGG_NOTCH_SIGNALING_PATHWAY [47]	Notch signaling pathway	6	2.09 e^{-3}	1.08 e^{-2}
KEGG_NEUROACTIVE_LIGAND_RECEPTOR_INTER TERACTION [272]	Neuroactive ligand-receptor interaction	17	2.23 e^{-3}	1.12 e^{-2}
KEGG_GLIOMA [65]	Glioma	7	2.47 e^{-3}	1.21 e^{-2}
KEGG_GLYCEROLIPID_METABOLISM [49]	Glycerolipid metabolism	6	2.59 e^{-3}	1.24 e^{-2}
KEGG_CHEMOKINE_SIGNALING_PATHWAY [189]	Chemokine signaling pathway	13	3.14 e^{-3}	1.44 e^{-2}
KEGG_GLYCOSAMINOGLYCAN_BIOSYNTHESIS_CH _CHONDROITIN_SULFATE [22]	Glycosaminoglycan biosynthesis - chondroitin sulfate	4	3.18 e^{-3}	1.44 e^{-2}
KEGG_PROSTATE_CANCER [89]	Prostate cancer	8	3.9 e^{-3}	1.73 e^{-2}
KEGG_VIBRIO_CHOLERAE_INFECTION [54]	Vibrio cholerae infection	6	4.25 e^{-3}	1.84 e^{-2}
KEGG_TIGHT_JUNCTION [132]	Tight junction	10	4.62 e^{-3}	1.95 e^{-2}
KEGG_ENDOCYTOSIS [181]	Endocytosis	12	5.93 e^{-3}	2.42 e^{-2}

KEGG_NATURAL_KILLER_CELL_MEDIATED_CYTOYTOTOXICITY [137]	Natural killer cell mediated cytotoxicity	10	$5.99 e^{-3}$	$2.42 e^{-2}$
KEGG_GLYCEROPHOSPHOLIPID_METABOLISM [77]	Glycerophospholipid metabolism	7	$6.37 e^{-3}$	$2.52 e^{-2}$
KEGG_GLYCOSPHINGOLIPID_BIOSYNTHESIS_GA_GANGLIO_SERIES [15]	Glycosphingolipid biosynthesis - ganglio series	3	$8.12 e^{-3}$	$3.15 e^{-2}$
KEGG_T_CELL_RECEPTOR_SIGNALING_PATHWAY [108]	T cell receptor signaling pathway	8	$1.22 e^{-2}$	$4.63 e^{-2}$

Supplementary Table 10. The 10 KEGG pathways overrepresented by genes in Kinship model from discovery sample with FDR< 0.05.

Gene set names[# Genes (K)]	Description	#genes in overlap(K)	P-value	FDR q-value
KEGG_AXON_GUIDANCE [129]	Axon guidance	13	$3.41\ e^{-5}$	$6.35\ e^{-3}$
KEGG_PPAR_SIGNALING_PATHWAY [69]	PPAR signaling pathway	9	$7.43\ e^{-5}$	$6.91\ e^{-3}$
KEGG_CHEMOKINE_SIGNALING_PATHWAY [189]	Chemokine signaling pathway	14	$4.67\ e^{-4}$	$2.89\ e^{-2}$
KEGG_FOCAL_ADHESION [199]	Focal adhesion	14	$7.77\ e^{-4}$	$3.61\ e^{-2}$
KEGG_ECM_RECEPTOR_INTERACTION [84]	ECM-receptor interaction	8	$1.57\ e^{-3}$	$4.62\ e^{-2}$
KEGG_CYSTEINE_AND_METHIONINE_METABOLIS LISM [34]	Cysteine and methionine metabolism	5	$1.76\ e^{-3}$	$4.62\ e^{-2}$
KEGG_WNT_SIGNALING_PATHWAY [150]	Wnt signaling pathway	11	$1.98\ e^{-3}$	$4.62\ e^{-2}$
KEGG_T_CELL_RECEPTOR_SIGNALING_PATHWAY [108]	T cell receptor signaling pathway	9	$2.09\ e^{-3}$	$4.62\ e^{-2}$
KEGG_TIGHT_JUNCTION [132]	Tight junction	10	$2.46\ e^{-3}$	$4.62\ e^{-2}$
KEGG_AMYOTROPHIC_LATERAL_SCLEROSIS_ALS [53]	Amyotrophic lateral sclerosis (ALS)	6	$2.49\ e^{-3}$	$4.62\ e^{-2}$

Supplementary Table 11. The 12 KEGG pathways overrepresented by genes in LME model from discovery sample with FDR< 0.05.

Gene set names[# Genes (K)]	Description	#genes in overlap(K)	P-value	FDR q-value
KEGG_AXON_GUIDANCE [129]	Axon guidance	13	3.01 e^{-5}	5.6 e^{-3}
KEGG_PPAR_SIGNALING_PATHWAY [69]	PPAR signaling pathway	9	6.77 e^{-5}	6.29 e^{-3}
KEGG_CHEMOKINE_SIGNALING_PATHWAY [189]	Chemokine signaling pathway	14	4.14 e^{-4}	2.57 e^{-2}
KEGG_FOCAL_ADHESION [199]	Focal adhesion	14	6.91 e^{-4}	3.21 e^{-2}
KEGG_ECM_RECEPTOR_INTERACTION [84]	ECM-receptor interaction	8	1.45 e^{-3}	4.35 e^{-2}
KEGG_CYSTEINE_AND_METHIONINE_METABOLIS LISM [34]	Cysteine and methionine metabolism	5	1.67 e^{-3}	4.35 e^{-2}
KEGG_WNT_SIGNALING_PATHWAY [150]	Wnt signaling pathway	11	1.8 e^{-3}	4.35 e^{-2}
KEGG_T_CELL_RECEPTOR_SIGNALING_PATHWAY [108]	T cell receptor signaling pathway	9	1.92 e^{-3}	4.35 e^{-2}
KEGG_TIGHT_JUNCTION [132]	Tight junction	10	2.25 e^{-3}	4.35 e^{-2}
KEGG_AMYOTROPHIC_LATERAL_SCLEROSIS_ALS [53]	Amyotrophic lateral sclerosis (ALS)	6	2.34 e^{-3}	4.35 e^{-2}
KEGG_LEUKOCYTE_TRANSENDOTHELIAL_MIGRAT RATION [116]	Leukocyte transendothelial migration	9	3.13 e^{-3}	4.92 e^{-2}
KEGG_B_CELL_RECEPTOR_SIGNALING_PATHWAY [75]	B cell receptor signaling pathway	7	3.18 e^{-3}	4.92 e^{-2}

Supplementary Table 12. The 35 KEGG pathways overrepresented by genes from GCC in replication sample with FDR < 0.05.

Gene Set Name [# Genes (K)]	Description	# Genes in Overlap (k)	p-value	FDR q-value
KEGG_PATHWAYS_IN_CANCER [325]	Pathways in cancer	34	$9.48\ e^{-12}$	$1.76\ e^{-9}$
KEGG_ARRHYTHMOGENIC_RIGHT_VENTRICULAR_AR_CARDIOMYOPATHY_ARVC [74]	Arrhythmogenic right ventricular cardiomyopathy (ARVC)	16	$7\ e^{-11}$	$6.51\ e^{-9}$
KEGG_AXON_GUIDANCE [129]	Axon guidance	19	$1.26\ e^{-9}$	$7.79\ e^{-8}$
KEGG_CALCIUM_SIGNALING_PATHWAY [177]	Calcium signaling pathway	22	$1.74\ e^{-9}$	$8.11\ e^{-8}$
KEGG_FOCAL_ADHESION [199]	Focal adhesion	21	$7.39\ e^{-8}$	$2.75\ e^{-6}$
KEGG_VASCULAR_SMOOTH_MUSCLE_CONTRACTIOTION [115]	Vascular smooth muscle contraction	15	$3.5\ e^{-7}$	$1.08\ e^{-5}$
KEGG_DILATED_CARDIOMYOPATHY [90]	Dilated cardiomyopathy	13	$6.39\ e^{-7}$	$1.7\ e^{-5}$
KEGG_HYPERTROPHIC_CARDIOMYOPATHY_HCM [83]	Hypertrophic cardiomyopathy (HCM)	12	$1.7\ e^{-6}$	$3.95\ e^{-5}$
KEGG_ECM_RECEPTOR_INTERACTION [84]	ECM-receptor interaction	12	$1.94\ e^{-6}$	$4\ e^{-5}$
KEGG_TYPE_II_DIABETES_MELLITUS [47]	Type II diabetes mellitus	8	$2.72\ e^{-5}$	$5.06\ e^{-4}$
KEGG_NEUROACTIVE_LIGAND_RECEPTOR_INTERTERACTION [272]	Neuroactive ligand-receptor interaction	20	$3.78\ e^{-5}$	$6.39\ e^{-4}$
KEGG_CELL_ADHESION_MOLECULES_CAMS [133]	Cell adhesion molecules (CAMs)	13	$4.95\ e^{-5}$	$7.68\ e^{-4}$
KEGG_GNRH_SIGNALING_PATHWAY [101]	GnRH signaling pathway	11	$6.95\ e^{-5}$	$9.95\ e^{-4}$
KEGG_PHOSPHATIDYLINOSITOL_SIGNALING_SY_SYSTEM [76]	Phosphatidylinositol signaling system	9	$1.65\ e^{-4}$	$2.19\ e^{-3}$
KEGG_PURINE_METABOLISM [159]	Purine metabolism	13	$3\ e^{-4}$	$3.72\ e^{-3}$
KEGG_SMALL_CELL_LUNG_CANCER [84]	Small cell lung cancer	9	$3.54\ e^{-4}$	$4.11\ e^{-3}$
KEGG_LONG_TERM_DEPRESSION [70]	Long-term depression	8	$4.82\ e^{-4}$	$5.07\ e^{-3}$
KEGG_CHEMOKINE_SIGNALING_PATHWAY [189]	Chemokine signaling pathway	14	$4.91\ e^{-4}$	$5.07\ e^{-3}$
KEGG_HEDGEHOG_SIGNALING_PATHWAY [56]	Hedgehog signaling pathway	7	$6.25\ e^{-4}$	$6.12\ e^{-3}$
KEGG_MAPK_SIGNALING_PATHWAY [267]	MAPK signaling pathway	17	$7.44\ e^{-4}$	$6.92\ e^{-3}$
KEGG_CARDIAC_MUSCLE_CONTRACTION [78]	Cardiac muscle contraction	8	$9.96\ e^{-4}$	$8.82\ e^{-3}$
KEGG_REGULATION_OF_ACTIN_CYTOSKELETON [213]	Regulation of actin cytoskeleton	14	$1.56\ e^{-3}$	$1.32\ e^{-2}$
KEGG_GLYCEROLIPID_METABOLISM [49]	Glycerolipid metabolism	6	$1.7\ e^{-3}$	$1.37\ e^{-2}$
KEGG_GAP_JUNCTION [90]	Gap junction	8	$2.51\ e^{-3}$	$1.9\ e^{-2}$
KEGG_TIGHT_JUNCTION [132]	Tight junction	10	$2.55\ e^{-3}$	$1.9\ e^{-2}$
KEGG_ADHERENS_JUNCTION [73]	Adherens junction	7	$3\ e^{-3}$	$2.12\ e^{-2}$
KEGG_BASAL_CELL_CARCINOMA [55]	Basal cell carcinoma	6	$3.08\ e^{-3}$	$2.12\ e^{-2}$
KEGG_NICOTINATE_AND_NICOTINAMIDE_METABTABOLISM [24]	Nicotinate and nicotinamide metabolism	4	$3.26\ e^{-3}$	$2.17\ e^{-2}$
KEGG_ALZHEIMERS_DISEASE [165]	Alzheimer's disease	11	$4.28\ e^{-3}$	$2.75\ e^{-2}$
KEGG_MELANOGENESIS [101]	Melanogenesis	8	$5.09\ e^{-3}$	$3.16\ e^{-2}$
KEGG_WNT_SIGNALING_PATHWAY [150]	Wnt signaling pathway	10	$6.3\ e^{-3}$	$3.78\ e^{-2}$
KEGG_O_GLYCAN_BIOSYNTHESIS [30]	O-Glycan biosynthesis	4	$7.44\ e^{-3}$	$4.3\ e^{-2}$
KEGG_ERBB_SIGNALING_PATHWAY [87]	ErbB signaling pathway	7	$7.87\ e^{-3}$	$4.3\ e^{-2}$
KEGG_HEMATOPOIETIC_CELL_LINEAGE [87]	Hematopoietic cell lineage	7	$7.87\ e^{-3}$	$4.3\ e^{-2}$
KEGG_ADIPOCYTOKINE_SIGNALING_PATHWAY [67]	Adipocytokine signaling pathway	6	$8.15\ e^{-3}$	$4.33\ e^{-2}$

Supplementary Table 13. The 16 KEGG pathways overrepresented by genes in Kinship model in replication sample with FDR < 0.05.

Gene Set Name [# Genes (K)]	Description	# Genes in Overlap (k)	p-value	FDR q-value
KEGG_PATHWAYS_IN_CANCER [325]	Pathways in cancer	28	7.2 e^{-8}	1.34 e^{-5}
KEGG_MAPK_SIGNALING_PATHWAY [267]	MAPK signaling pathway	23	1.04 e^{-6}	9.63 e^{-5}
KEGG_T_CELL_RECEPTOR_SIGNALING_PATHWAY [108]	T cell receptor signaling pathway	13	6.57 e^{-6}	4.07 e^{-4}
KEGG_ARRHYTHMOGENIC_RIGHT_VENTRICULAR_AR_CARDIOMYOPATHY_ARVC [74]	Arrhythmogenic right ventricular cardiomyopathy (ARVC)	10	2.74 e^{-5}	1.06 e^{-3}
KEGG_FOCAL_ADHESION [199]	Focal adhesion	17	2.85 e^{-5}	1.06 e^{-3}
KEGG_B_CELL_RECEPTOR_SIGNALING_PATHWAY [75]	B cell receptor signaling pathway	9	1.75 e^{-4}	5.43 e^{-3}
KEGG_WNT_SIGNALING_PATHWAY [150]	Wnt signaling pathway	13	2.09 e^{-4}	5.56 e^{-3}
KEGG_NATURAL_KILLER_CELL_MEDIATED_CYTOYTOTOXICITY [137]	Natural killer cell mediated cytotoxicity	12	3.28 e^{-4}	7.63 e^{-3}
KEGG_CELL_CYCLE [124]	Cell cycle	11	5.16 e^{-4}	1.07 e^{-2}
KEGG_MELANOMA [71]	Melanoma	8	6.12 e^{-4}	1.14 e^{-2}
KEGG_OLFACTORY_TRANSDUCTION [389]	Olfactory transduction	22	9.05 e^{-4}	1.53 e^{-2}
KEGG_ECM_RECEPTOR_INTERACTION [84]	ECM-receptor interaction	8	1.85 e^{-3}	2.87 e^{-2}
KEGG_PARKINSONS_DISEASE [128]	Parkinson's disease	10	2.38 e^{-3}	3.41 e^{-2}
KEGG_METABOLISM_OF_XENOBIOTICS_BY_CYTOYTOCHROME_P450 [70]	Metabolism of xenobiotics by cytochrome P450	7	2.66 e^{-3}	3.54 e^{-2}
KEGG_DRUG_METABOLISM_CYTOCHROME_P450 [72]	Drug metabolism - cytochrome P450	7	3.13 e^{-3}	3.88 e^{-2}
KEGG_ADHERENS_JUNCTION [73]	Adherens junction	7	3.38 e^{-3}	3.93 e^{-2}

Supplementary Table 14. 12 KEGG pathways overrepresented by genes in LME model in replication sample with FDR <0.05.

Gene Set Name [# Genes (K)]	Description	# Genes in Overlap (k)	p-value	FDR q-value
KEGG_PATHWAYS_IN_CANCER [325]	Pathways in cancer	24	3.26 e^{-6}	6.07 e^{-4}
KEGG_MAPK_SIGNALING_PATHWAY [267]	MAPK signaling pathway	20	1.7 e^{-5}	1.15 e^{-3}
KEGG_T_CELL_RECEPTOR_SIGNALING_PATHWAY [108]	T cell receptor signaling pathway	12	1.86 e^{-5}	1.15 e^{-3}
KEGG_FOCAL_ADHESION [199]	Focal adhesion	16	4.98 e^{-5}	2.32 e^{-3}
KEGG_ARRHYTHMOGENIC_RIGHT_VENTRICULAR_AR_CARDIOMYOPATHY_ARVC [74]	Arrhythmogenic right ventricular cardiomyopathy (ARVC)	9	1.01 e^{-4}	3.75 e^{-3}
KEGG_WNT_SIGNALING_PATHWAY [150]	Wnt signaling pathway	12	4.41 e^{-4}	1.37 e^{-2}
KEGG_B_CELL_RECEPTOR_SIGNALING_PATHWAY [75]	B cell receptor signaling pathway	8	6 e^{-4}	1.6 e^{-2}
KEGG_PARKINSONS_DISEASE [128]	Parkinson's disease	10	1.54 e^{-3}	3.59 e^{-2}
KEGG_METABOLISM_OF_XENOBIOTICS_BY_CYTOYTOCHROME_P450 [70]	Metabolism of xenobiotics by cytochrome P450	7	1.91 e^{-3}	3.94 e^{-2}
KEGG_DRUG_METABOLISM_CYTOCHROME_P450 [72]	Drug metabolism - cytochrome P450	7	2.25 e^{-3}	3.95 e^{-2}
KEGG_ADHERENS_JUNCTION [73]	Adherens junction	7	2.43 e^{-3}	3.95 e^{-2}
KEGG_NATURAL_KILLER_CELL_MEDIATED_CYTOYTOTOXICITY [137]	Natural killer cell mediated cytotoxicity	10	2.55 e^{-3}	3.95 e^{-2}

Supplementary Table 15. The 28 KEGG pathways overrepresented by overlapped genes in GCC models in both studies with FDR < 0.05.

Gene Set Name [# Genes (K)]	Description	# Genes in Overlap (k)	p-value	FDR q-value
KEGG_ARRHYTHMOGENIC_RIGHT_VENTRICULAR_AR_CARDIOMYOPATHY_ARVC [74]	Arrhythmogenic right ventricular cardiomyopathy (ARVC)	10	2.83 e^{-8}	2.11 e^{-6}
KEGG_PATHWAYS_IN_CANCER [325]	Pathways in cancer	19	3.4 e^{-8}	2.11 e^{-6}
KEGG_CALCIUM_SIGNALING_PATHWAY [177]	Calcium signaling pathway	14	5.51 e^{-8}	2.56 e^{-6}
KEGG_AXON_GUIDANCE [129]	Axon guidance	12	8.33 e^{-8}	3.1 e^{-6}
KEGG_FOCAL_ADHESION [199]	Focal adhesion	13	1.46 e^{-6}	4.53 e^{-5}
KEGG_HYPERTROPHIC_CARDIOMYOPATHY_HCM [83]	Hypertrophic cardiomyopathy (HCM)	8	9.37 e^{-6}	2.49 e^{-4}
KEGG_DILATED_CARDIOMYOPATHY [90]	Dilated cardiomyopathy	8	1.71 e^{-5}	3.97 e^{-4}
KEGG_CELL_ADHESION_MOLECULES_CAMS [133]	Cell adhesion molecules (CAMs)	9	4.58 e^{-5}	9.47 e^{-4}
KEGG_ECM_RECEPTOR_INTERACTION [84]	ECM-receptor interaction	7	8.71 e^{-5}	1.62 e^{-3}
KEGG_LONG_TERM_DEPRESSION [70]	Long-term depression	6	2.39 e^{-4}	3.71 e^{-3}
KEGG_LONG_TERM_POTENTIATION [70]	Long-term potentiation	6	2.39 e^{-4}	3.71 e^{-3}
KEGG_GNRH_SIGNALING_PATHWAY [101]	GnRH signaling pathway	7	2.76 e^{-4}	3.85 e^{-3}
KEGG_TYPE_II_DIABETES_MELLITUS [47]	Type II diabetes mellitus	5	2.9 e^{-4}	3.85 e^{-3}
KEGG_BASAL_CELL_CARCINOMA [55]	Basal cell carcinoma	5	6.06 e^{-4}	7.45 e^{-3}
KEGG_SMALL_CELL_LUNG_CANCER [84]	Small cell lung cancer	6	6.41 e^{-4}	7.45 e^{-3}
KEGG_GAP_JUNCTION [90]	Gap junction	6	9.22 e^{-4}	1.01 e^{-2}
KEGG_TIGHT_JUNCTION [132]	Tight junction	7	1.36 e^{-3}	1.41 e^{-2}
KEGG_REGULATION_OF_ACTIN_CYTOSKELETON [213]	Regulation of actin cytoskeleton	9	1.48 e^{-3}	1.45 e^{-2}
KEGG_ADHERENS_JUNCTION [73]	Adherens junction	5	2.18 e^{-3}	2.03 e^{-2}
KEGG_NEUROACTIVE_LIGAND_RECEPTOR_INTERTERACTION [272]	Neuroactive ligand-receptor interaction	10	2.32 e^{-3}	2.05 e^{-2}
KEGG_PHOSPHATIDYLINOSITOL_SIGNALING_SY_SYSTEM [76]	Phosphatidylinositol signaling system	5	2.6 e^{-3}	2.2 e^{-2}
KEGG_NOTCH_SIGNALING_PATHWAY [47]	Notch signaling pathway	4	2.76 e^{-3}	2.23 e^{-2}
KEGG_CARDIAC_MUSCLE_CONTRACTION [78]	Cardiac muscle contraction	5	2.91 e^{-3}	2.26 e^{-2}
KEGG_ERBB_SIGNALING_PATHWAY [87]	ErbB signaling pathway	5	4.66 e^{-3}	3.4 e^{-2}
KEGG_ALZHEIMERS_DISEASE [165]	Alzheimer's disease	7	4.75 e^{-3}	3.4 e^{-2}
KEGG_HEDGEHOG_SIGNALING_PATHWAY [56]	Hedgehog signaling pathway	4	5.2 e^{-3}	3.58 e^{-2}
KEGG_MAPK_SIGNALING_PATHWAY [267]	MAPK signaling pathway	9	6.57 e^{-3}	4.36 e^{-2}
